# Prediction of vancomycin-resistant phenotype in ICU-onset sterile-site *Enterococcus*-positive cultures: development and pseudo-temporal validation of a clinical prediction model using MIMIC-IV

**DOI:** 10.3389/fcimb.2026.1935284

**Published:** 2026-10-02

**Authors:** Wenhao Sun, Xiao-lan Gu, Qingmei Ma, Jinxia Hou, Yanbin Chang

**Affiliations:** 1 School of Information Engineering and Artificial Intelligence, Lanzhou University of Finance and Economics, Lanzhou, China; 2 Department of Clinical Laboratory, Gansu Provincial Hospital, Lanzhou, Gansu, China

**Keywords:** antimicrobial stewardship, clinical prediction model, intensive care unit, MIMIC-IV, temporal validation, vancomycin-resistant *Enterococcus*

## Abstract

**Background:**

Vancomycin-resistant enterococci challenge intensive care. During the interval between organism identification and final phenotypic antimicrobial susceptibility testing, clinicians must balance delayed effective anti-VRE therapy against unnecessary anti-VRE exposure. We aimed to develop and pseudo-temporally validate a model for the vancomycin-resistance phenotype of the index isolate; the model does not determine whether the isolate represents clinically significant infection.

**Methods:**

In this retrospective MIMIC-IV cohort, adult ICU admissions with a sterile-site *Enterococcus*-positive culture obtained more than 48 hours after ICU admission were assigned by de-identified anchor-year group to development (groups labelled 2008–2016; n = 552) or pseudo-temporal validation (2017–2022; n = 259). Predictors were restricted to information available by specimen collection. Elastic Net with bootstrap stability screening retained 19 of 141 candidates; four tree-based models were comparators.

**Results:**

Among 811 admissions (507 resistant isolates; prevalence, 62.5%), Elastic Net achieved a validation AUROC of 0.835 (95% CI, 0.778–0.889) and calibration slope of 0.91 (0.71–1.18). Tree-model AUROCs were 0.842–0.855; non-significant pairwise DeLong tests were evidence insufficient to detect differences, not evidence of equivalence. Removing both species indicators reduced AUROC to 0.657 (DeLong *P* < 0.001). Development-OOF-fitted Platt scaling improved validation slopes for Random Forest, XGBoost, and CatBoost from 1.41, 2.41, and 1.46 to 0.94, 1.02, and 1.01. At the Youden index threshold, Elastic Net sensitivity was 0.884 and specificity was 0.690. In exploratory decision-curve analysis, its net benefit did not exceed treat-all at thresholds 0.10 or 0.20; the paired difference was 0.044 (95% CI, 0.014–0.071) at 0.30.

**Conclusions:**

The model predicts isolate resistance phenotype, not adjudicated infection, and is not a stand-alone treatment rule. These estimates are specific to a single-center cohort with high VRE prevalence. Independent external validation, local recalibration, clinical adjudication, and periodic updating are prerequisites for use.

## 1 Introduction

Antimicrobial resistance (AMR) is a major threat to global health, with an estimated 4.95 million deaths associated with bacterial AMR in 2019 (Antimicrobial Resistance Collaborators, 2022). *Enterococcus faecium*, a member of the ESKAPE pathogen group (*E. faecium*, *Staphylococcus aureus*, *Klebsiella pneumoniae*, *Acinetobacter baumannii*, *Pseudomonas aeruginosa*, and *Enterobacter* species), is particularly important because vancomycin-resistant enterococci (VRE) remain a persistent challenge in critical care settings (De Oliveira et al., 2020). In the United States, the CDC 2019 Antibiotic Resistance Threats Report estimated approximately 54,500 hospital-onset VRE infections and 5,400 associated deaths in 2017, at a healthcare cost of USD 539 million (Centers for Disease Control and Prevention, 2019). Subsequent CDC surveillance documented that six monitored types of hospital-onset antimicrobial-resistant infection, including VRE, increased by approximately 20% in aggregate during the COVID-19 pandemic and remained collectively above pre-pandemic levels through 2022 (Centers for Disease Control and Prevention, 2024). Within the intensive care unit (ICU), where disease severity, indwelling-device density, and cumulative antibiotic pressure converge, enterococcal bloodstream infections carry substantial mortality that increases when empirical antimicrobial therapy does not cover the eventual resistance phenotype (Suppli et al., 2011; Cairns et al., 2023), and are associated with prolonged length of stay and higher healthcare costs (Song et al., 2003). A recent multicenter US analysis of 150,948 ICU admissions across 85 hospitals reported that vancomycin-resistant strains accounted for 69.8% of enterococcal ICU-onset bloodstream infections and were independently associated with mortality (adjusted OR 6.0; 95% CI, 1.7-21.3) (Gouel-Cheron et al., 2022). Evidence remains limited on sociodemographic inequalities in VRE risk and on whether prediction-model performance differs across demographic groups.

Rapid diagnostic technologies, including matrix-assisted laser desorption/ionization time-of-flight mass spectrometry (MALDI-TOF MS) and multiplex polymerase chain reaction, have substantially shortened the time to pathogen identification, often resolving genus- or species-level identification within hours of culture positivity (Vasala et al., 2020). However, genotype-based resistance detection cannot yet reliably replace phenotypic antimicrobial susceptibility testing (AST): phenotypic expression of *van* genes is regulatory and does not always mirror genotype (De Oliveira et al., 2020), and organisms may harbor resistance mechanisms not captured by targeted molecular panels. Phenotypic AST therefore remains the reference standard for clinical decision-making, but final susceptibility results typically become available substantially later than initial organism identification. This creates a pre-susceptibility window in which *Enterococcus* has been detected and routinely collected clinical and microbiological information is available, while the vancomycin-resistance phenotype remains pending. Within this window, clinicians face a therapeutic dilemma with costs on both sides. Delaying effective anti-VRE therapy until AST finalization can worsen outcomes when the isolate ultimately proves resistant, as enterococcal bloodstream-infection studies show higher mortality when empirical antimicrobial therapy does not cover the eventual resistance phenotype (Suppli et al., 2011; Cairns et al., 2023). Conversely, preemptive administration of anti-VRE agents (linezolid, daptomycin) to all patients with sterile-site *Enterococcus*-positive cultures exposes patients with vancomycin-susceptible isolates to unnecessary drug toxicity, including linezolid-associated thrombocytopenia (Cazavet et al., 2020), contributes to selection pressure favoring resistance, and increases pharmacy costs. An individualized risk-stratification tool that estimates the probability of vancomycin resistance during the pre-susceptibility window could help clinicians weigh these asymmetric costs at the bedside.

Several prediction models have addressed VRE or multidrug-resistant organism (MDRO) risk in hospitalized patients, but three structural limitations recur across this body of work. First, target populations and clinical scenarios are misaligned with an ICU-onset sterile-site culture-positive presentation. Existing models have been developed for VRE identification at ICU admission (Yoon et al., 2012), for prediction of vancomycin resistance in enterococcal bacteremia based on prior rectal-screening results (Butler-Laporte et al., 2020), for VRE bloodstream infection in leukemia induction and hematopoietic stem-cell transplantation cohorts (Webb et al., 2017), where a model incorporating VRE colonization status achieved an AUROC of 0.84, and for colonization-persistence risk at readmission (Boeing et al., 2021). More recent efforts have used deep learning (Wang et al., 2023) or gradient-boosted trees (Li et al., 2024) to predict composite MDRO endpoints among ICU patients from electronic health record data. To our knowledge, no published model has been designed specifically to estimate the probability of vancomycin resistance when a sterile-site *Enterococcus* culture becomes positive during ICU stay and phenotypic AST remains pending. Second, most models have relied on random split-sample or internal cross-validation; of the reviewed studies, only the PREVENT score for VRE colonization persistence (Boeing et al., 2021) and a MALDI-TOF-based classification model for VRE *E. faecium* (Sun et al., 2026) have used pseudo-temporal validation, in which models are trained on earlier-period data and tested on later-period isolates. Pseudo-temporal validation matters for AMR prediction because resistance epidemiology drifts over time: surveillance data document longitudinal shifts in the relative prevalence of *vanA* versus *vanB* enterococcal clusters (Hammerum et al., 2024) and changes in VRE rates in European and US hospitals (Mendes et al., 2016). Third, routinely recorded culture-level information, such as specimen type and species-level identification, has not been consistently integrated with clinical variables in prediction models targeting this population, although Liu et al. reported that specimen type was a significant predictor in a model for linezolid-resistant *E. faecalis* (Liu et al., 2024), providing initial evidence that culture-level context carries predictive information in antimicrobial-resistance modeling.

We therefore aimed to develop and pseudo-temporally validate a clinical prediction model for vancomycin resistance among adult ICU patients with sterile-site *Enterococcus*-positive cultures obtained more than 48 hours after ICU admission. The study is reported in accordance with the TRIPOD+AI statement (Collins et al., 2024) and addresses one component of the clinical decision problem: the vancomycin-resistance phenotype of the index isolate before phenotypic AST is finalized. It does not model the separate question of whether that isolate represents clinically significant infection rather than colonization or contamination; both components would need to be considered in any deployment framework. Using demographic and clinical variables recorded by specimen collection, together with specimen source and species identification recorded for the index culture before incorporation of the vancomycin susceptibility result, we trained models in MIMIC-IV anchor-year groups labeled 2008–2016 and evaluated performance in groups labeled 2017-2022. A penalized logistic regression served as the interpretable reference model, and four tree-based algorithms were evaluated as comparators on a common predictor set, with joint assessment of discrimination, calibration, and exploratory decision curve analysis.

## 2 Materials and methods

### 2.1 Study design and data source

This was a retrospective cohort study using data from the Medical Information Mart for Intensive Care IV (MIMIC-IV, version 3.0) database. MIMIC-IV contains de-identified health data from patients admitted to Beth Israel Deaconess Medical Center (Boston, MA, USA) between 2008 and 2022. The database is accessible via PhysioNet (physionet.org) to credentialed users who have completed the Collaborative Institutional Training Initiative (CITI) human-subjects training. The creation of the MIMIC-IV database was approved by the institutional review boards of Beth Israel Deaconess Medical Center and the Massachusetts Institute of Technology; the requirement for individual patient consent was waived because the data are de-identified. No prospective study protocol was prepared, and this retrospective database study was not prospectively registered.

The study is reported in accordance with the TRIPOD+AI statement, which extends the Transparent Reporting of a Multivariable Prediction Model for Individual Prognosis or Diagnosis guideline to prediction models incorporating machine learning (Collins et al., 2024); the completed TRIPOD+AI and TRIPOD+AI for Abstracts reporting checklists are provided in **Supplementary Tables S17** and **S18**.

### 2.2 Study population

#### 2.2.1 Inclusion criteria

Adult patients (aged ≥ 18 years) admitted to Beth Israel Deaconess Medical Center between 2008 and 2022 were eligible if they had a recorded ICU stay and met all of the following criteria: (1) at least one clinical culture was obtained from a normally sterile body site (blood, peritoneal fluid, pleural fluid, cerebrospinal fluid, joint fluid, tissue, biopsy, dialysis fluid, aspirate, bone marrow, or fluid from other normally sterile body cavities); (2) the culture yielded *Enterococcus* spp.; (3) vancomycin susceptibility testing was performed on the isolate; and (4) the susceptibility result was interpretable as susceptible (S) or resistant (R) according to the clinical microbiology laboratory report. To focus on ICU-onset or ICU-persistent culture-positive episodes, eligible cultures were required to have been obtained more than 48 hours after ICU admission. Time from ICU admission to specimen collection was computed in hours from the recorded ICU admission time to the specimen collection time recorded in the MIMIC-IV microbiology events table.

#### 2.2.2 Exclusion criteria

We excluded, in the following sequence (**Figure 1**): (1) cultures from non-sterile sites, including respiratory specimens, urine, wound swabs, and stool surveillance or rectal screening specimens; (2) isolates from organisms other than *Enterococcus* spp.; (3) isolates without a vancomycin susceptibility test result; (4) isolates with intermediate (I) vancomycin susceptibility, because their clinical interpretation is ambiguous and they cannot be reliably classified as VRE or vancomycin-susceptible *Enterococcus* (VSE); (5) duplicate culture episodes, defined as cultures of the same specimen type yielding the same *Enterococcus* species within 14 days of a prior culture in the same admission—only the earliest culture in each episode was retained; (6) admissions without a recorded ICU stay; and (7) cultures obtained 48 hours or less after ICU admission. For ICU admissions with multiple remaining eligible culture episodes, only the first was retained as the index culture (**Figure 1**).

**Figure 1:**
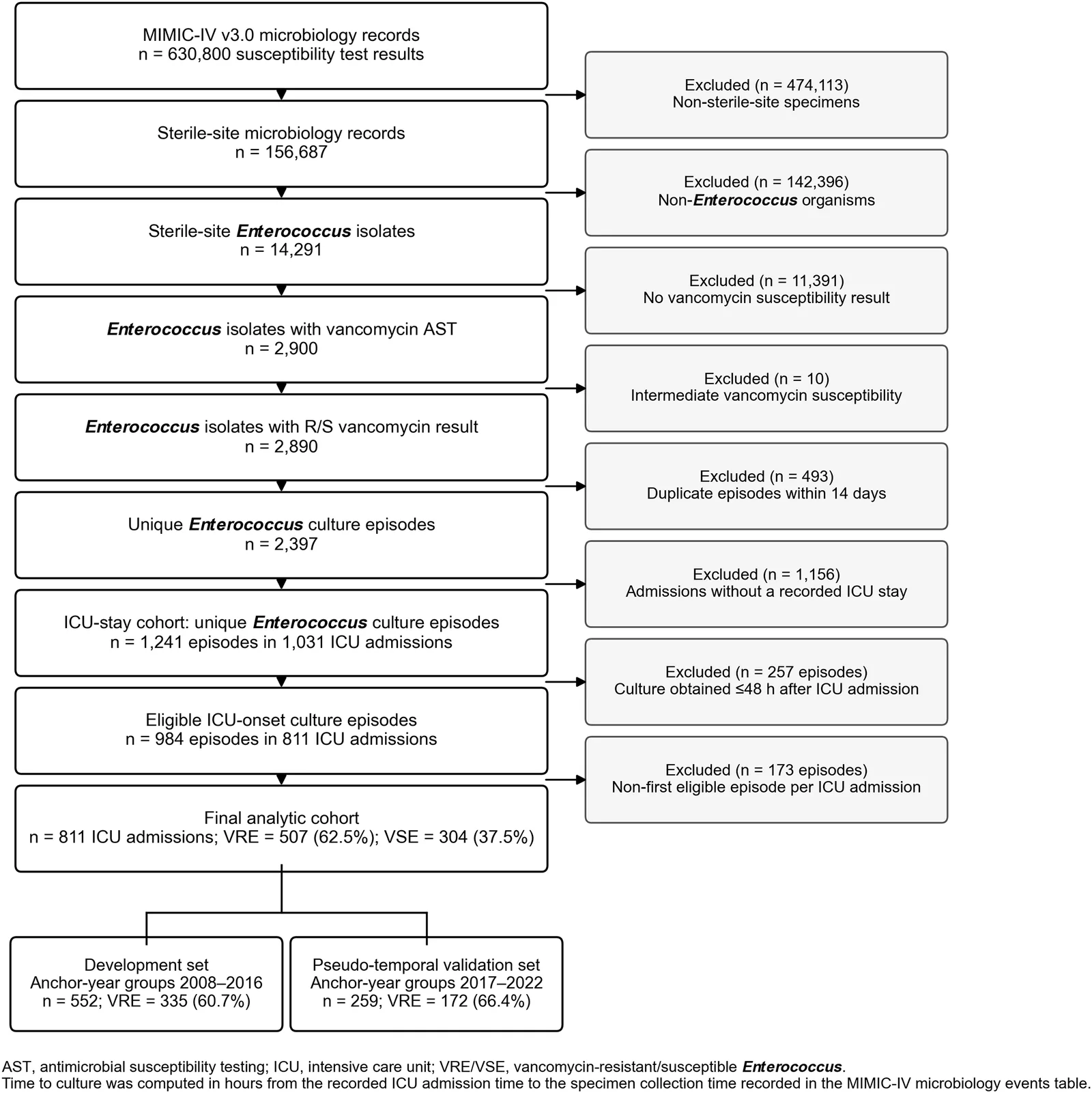
Study flow diagram for the construction of the analytic cohort. Adult ICU admissions in the Medical Information Mart for Intensive Care IV (MIMIC-IV, version 3.0) database with a sterile-site *Enterococcus*-positive culture obtained more than 48 hours after ICU admission were identified across anchor-year groups labelled 2008–2022. The diagram shows the sequential application of exclusion criteria: (1) cultures from non-sterile sites; (2) isolates from organisms other than *Enterococcus* spp.; (3) isolates without a vancomycin susceptibility test result; (4) isolates with intermediate vancomycin susceptibility; (5) duplicate culture episodes (defined as cultures of the same specimen type yielding the same *Enterococcus* species within 14 days of a prior culture in the same admission); (6) admissions without a recorded ICU stay; and (7) cultures obtained 48 hours or less after ICU admission. For ICU admissions with multiple eligible cultures, only the first was retained as the index culture. The final analytic cohort comprised 811 ICU admissions (507 VRE [62.5%] and 304 VSE [37.5%]). The cohort was split pseudo-temporally by anchor-year groups labelled 2008–2016 (n = 552; 335 VRE) for development and 2017–2022 (n = 259; 172 VRE) for pseudo-temporal validation. ICU, intensive care unit; VRE, vancomycin-resistant *Enterococcus*; VSE, vancomycin-susceptible *Enterococcus*.

#### 2.2.3 Unit of analysis, index culture, and analytical prediction point

The unit of analysis was the ICU admission. For ICU admissions with multiple eligible cultures after applying the criteria above, only the first was retained as the index culture to avoid repeated observations within the same ICU admission; this index culture was selected according to specimen collection time. The analytical prediction point was defined as assessment of an index culture identified as *Enterococcus*-positive before incorporation of the final vancomycin susceptibility result. This definition represents a pre-susceptibility modeling setting rather than reconstruction of an exact laboratory reporting time. To prevent information leakage from clinical changes after culture collection, clinical predictors (demographics, comorbidities, antimicrobial exposures, procedures, severity scores, vital signs, and laboratory values) were restricted to information available at or before the specimen collection time. Culture-related variables were used as recorded for the index culture; cultures without species-level identification were retained in an explicit “species not specified” category. The final vancomycin susceptibility result was used only to define the outcome and was not included as a predictor.

### 2.3 Outcome definition

The primary outcome was vancomycin resistance in the index *Enterococcus*-positive culture, as determined by the clinical microbiology laboratory and recorded in MIMIC-IV. Cultures classified as resistant (R) were defined as VRE; those classified as susceptible (S) were defined as VSE. Clinical significance was not adjudicated; the modeled outcome was the resistance phenotype of the recorded index isolate, not the occurrence or causal attribution of infection, treatment response, or patient prognosis. Distributions of the 19 final model predictors by VRE status are summarized in **Table 1**.

**Table 1:** Distribution of the 19 final model predictors by vancomycin-resistance outcome.

Variable	Overall (n = 811)	VSE (n = 304)	VRE (n = 507)	P value	SMD
Species					
*E. faecium*	428 (52.8)	56 (18.4)	372 (73.4)	<0.001	1.322
*E. faecalis*	128 (15.8)	113 (37.2)	15 (3.0)	<0.001	−0.945
Specimen source					
Tissue	142 (17.5)	77 (25.3)	65 (12.8)	<0.001	−0.322
Peritoneal fluid	110 (13.6)	40 (13.2)	70 (13.8)	0.833	0.019
Clinical severity					
SOFA score	8 (5–12)	7 (4–11)	9 (6–13)	<0.001	0.412
Comorbidity and clinical status					
Hyperlipidemia	248 (30.6)	100 (32.9)	148 (29.2)	0.272	−0.080
Mechanical ventilation	650 (80.1)	257 (84.5)	393 (77.5)	0.018	−0.180
Widowed	78 (9.6)	37 (12.2)	41 (8.1)	0.065	−0.136
Medication exposures					
Nephrotoxic agents	517 (63.7)	153 (50.3)	364 (71.8)	<0.001	0.451
Vancomycin	236 (29.1)	68 (22.4)	168 (33.1)	0.001	0.242
Rifamycin	97 (12.0)	15 (4.9)	82 (16.2)	<0.001	0.372
Sulfamethoxazole–trimethoprim	69 (8.5)	23 (7.6)	46 (9.1)	0.517	0.055
Corticosteroids	217 (26.8)	60 (19.7)	157 (31.0)	<0.001	0.260
Procedures					
Endoscopic procedure	443 (54.6)	142 (46.7)	301 (59.4)	<0.001	0.256
Renal replacement therapy procedure	259 (31.9)	70 (23.0)	189 (37.3)	<0.001	0.314
Skin and soft-tissue procedure	214 (26.4)	69 (22.7)	145 (28.6)	0.070	0.135
Percutaneous drainage	488 (60.2)	163 (53.6)	325 (64.1)	0.004	0.214
Laboratory values and temperature					
Aspartate aminotransferase (U/L)	53 (28–129)	47 (24.5–130)	55.5 (30–128)	0.094	−0.035
Temperature (°C)	36.8 (36.6–37.1)	36.8 (36.6–37.1)	36.8 (36.5–37.1)	0.195	−0.112

Values are median (interquartile range) or n (%). For each displayed species and specimen-source indicator, the P value compares presence versus absence of that level; no omnibus P value for the underlying multilevel variable is shown. Continuous variables were compared using the Wilcoxon rank-sum test and binary indicators using Fisher’s exact test. SMDs are the primary descriptors of difference magnitude.

SMD, standardized mean difference; SOFA, Sequential Organ Failure Assessment; U/L, units per liter; VRE, vancomycin-resistant *Enterococcus*; VSE, vancomycin-susceptible *Enterococcus*.

### 2.4 Predictors

Candidate predictors included demographic variables, comorbidities, antimicrobial exposures during the current ICU admission prior to specimen collection, invasive procedures and organ-support interventions, ICU severity scores (SOFA and SAPS II), and vital signs and laboratory measurements summarized over the first 24 hours of ICU admission. Demographic variables included marital status as a routinely recorded potential marker of social and care-pathway context, not as a presumed causal determinant of resistance. The first-24-hour window for severity scores, vital signs, and laboratory values captures baseline severity of illness at ICU admission, follows standard ICU severity-score conventions, and—because eligible cultures were required to have been obtained more than 48 hours after ICU admission—is by design earlier than the analytical prediction point. Culture-related variables—specimen source, *Enterococcus* species as recorded, and time from ICU admission to specimen collection—were included as components of the multivariable predictor set. Variables functioning as identifiers or used solely for pseudo-temporal splitting were excluded from the predictor set. Specimen-source categories and candidate-predictor distributions are summarized in **Supplementary Table S3**.

### 2.5 Pseudo-temporal split by anchor-year group

Data were split temporally by anchor-year group, defined as three-year intervals labelled 2008–2010, 2011–2013, 2014–2016, 2017–2019, and 2020–2022. MIMIC-IV uses de-identified anchor-year information; therefore, these intervals should be interpreted as database anchor-year groups rather than exact calendar-year labels for individual patients. Anchor-year groups labelled 2008–2016 were assigned to the development set, and those labelled 2017–2022 were held out as the pseudo-temporal validation set (**Figure 1**). This design simulates the intended deployment scenario in which a model trained on earlier anchor-year groups is applied to later groups, and reduces the optimistic bias that can arise from random cross-sectional splitting in a temporally evolving setting.

### 2.6 Missing data and preprocessing

Predictors were categorized as binary, continuous, or categorical. Binary variables (e.g., comorbidities, medication exposures, procedures) were imputed with the mode. Continuous variables (e.g., laboratory values, vital signs, severity scores) were imputed using iterative imputation (single imputation without posterior draws; maximum 20 iterations; initial strategy, median). Source-reported categorical levels for specimen source and *Enterococcus* species were preserved before one-hot encoding; specifically, cultures reported as *Enterococcus* without further species specification were retained as an explicit “species not specified” level rather than imputed to *E. faecium* or another named species. True missing categorical values, if present after source-level harmonization, were imputed with the mode and subsequently one-hot encoded. The initial encoder retained indicators for all observed levels; feature selection subsequently retained only the *E. faecium* and *E. faecalis* indicators for species and the peritoneal-fluid and tissue indicators for specimen source. Consequently, in the final model, the species reference group comprised “other *Enterococcus* species” and “*Enterococcus* spp. (species not specified),” and the specimen-source reference group comprised all observed source levels other than peritoneal fluid and tissue (aspirate, biopsy, blood culture, myco/F lytic blood culture, cerebrospinal fluid, dialysis fluid, fluid received in blood-culture bottles, other fluid, foreign body, and pleural fluid). For each continuous variable with any missing values, a binary missing indicator was generated and entered into the candidate predictor pool on the same basis as other binary variables, allowing the selection procedure to evaluate the potential informativeness of missingness. All preprocessing steps—including imputation, one-hot encoding, and missing-indicator construction—were fitted on the development set and then applied to the pseudo-temporal validation set to prevent information leakage.

### 2.7 Feature selection

Feature selection was performed exclusively within the development set. Elastic Net logistic regression (elastic-net penalty; solver, SAGA; maximum 20,000 iterations) was used as the selector. The regularization strength *C* (50 log-spaced values from 10^-^³ to 10²) and the L1/L2 mixing parameter *l1_ratio* (0.1, 0.3, 0.5, 0.7, 0.9, 0.95) were tuned by grid search with leave-one-year-group-out cross-validation (time-block CV) to preserve the temporal structure of the data. When time-block CV produced fewer than two valid folds, stratified *k*-fold cross-validation was available as a fallback; this fallback was not triggered because all three development time blocks yielded valid folds.

The best Elastic Net model was then refitted on the full development set. Bootstrap stability analysis (100 resamples of the development set) was performed to estimate each candidate predictor’s selection frequency and sign consistency. Predictors with a selection frequency ≥ 0.50 and a non-zero coefficient in the final Elastic Net model were considered stable; sign consistency was reported as a descriptive stability metric. Before construction of the final feature set, development-set support was assessed to prevent sparse binary predictors from producing separation-prone coefficients. Binary and one-hot encoded predictors were eligible only when at least 20 admissions had the predictor coded as present (=1) in the development set and every cell of the predictor (0/1) by outcome (VSE/VRE) 2×2 table contained at least 5 observations. Continuous predictors required at least 5 unique development-set values and non-zero variance. The pseudo-temporal validation set was not used for this eligibility assessment. To preserve model parsimony and ensure at least 10 observations in the smaller outcome class per candidate final predictor, a prespecified maximum of 20 final predictors was imposed. Because VRE was coded as the event, the conventional events-per-variable ratio (EPV) was calculated using the number of VRE outcomes; the corresponding ratio based on the smaller VSE outcome class was reported separately and was not termed EPV. Eligible stable predictors were ranked by bootstrap selection frequency and absolute Elastic Net coefficient magnitude; 19 met all criteria and were retained. The complete eligibility audit is reported in **Supplementary Table S5**.

### 2.8 Sample size considerations

All 811 eligible admissions were included, and the temporally earlier 552 admissions were available for model development; no formal *a priori* sample-size calculation was performed because the study used all eligible observations in a fixed retrospective data source rather than accruing participants to a target. In the development set, 335 VRE events and 217 VSE outcomes were available for the 19 retained predictors, corresponding to a conventional EPV of 17.63 and 11.42 observations per predictor in the smaller outcome class. Penalization, bootstrap stability assessment, support screening, and a prespecified cap of 20 final predictors were used to constrain model complexity. These measures do not eliminate the uncertainty arising from the development sample size. This uncertainty is considered when interpreting coefficient stability and motivates the need for larger external validation datasets.

### 2.9 Model development

Five classification models were trained on the same 19 final predictors using the development set. This common predictor set was selected by the Elastic Net procedure described above and was held fixed across models to standardize the input information used for algorithm comparison:

Elastic Net logistic regression served as the interpretable reference model, providing odds ratios and directly interpretable coefficients; Random Forest, XGBoost, LightGBM, and CatBoost were included as machine-learning comparators.

No single model was designated as the sole primary model *a priori*. Elastic Net logistic regression was specified as the interpretable reference, and the four tree-based models were specified as machine-learning comparators. Pairwise pseudo-temporal-validation AUROC comparisons were used to assess whether any comparator showed statistically distinguishable discrimination, not to guide model tuning.

Hyperparameters were tuned within the development set using time-block CV where feasible, with stratified *k*-fold CV (3 folds) as a fallback; this fallback was not triggered in this study. Grid search was used for Elastic Net (*C*: 50 log-spaced values; *l1_ratio*: 0.1, 0.3, 0.5, 0.7, 0.9, 0.95) and Random Forest. Randomized search was used for XGBoost (20 iterations), LightGBM (20 iterations), and CatBoost (15 iterations). The full hyperparameter search spaces and final selected values are reported in **Supplementary Table S15** and archived with the model artifacts. The best hyperparameters were selected by cross-validated AUROC. All models were then refitted on the full development set for final evaluation.

For Elastic Net, continuous predictors in the final model matrix were standardized (zero mean, unit variance) within the development-set pipeline; binary and one-hot encoded predictors were retained on their original 0/1 scale, and tree-based models were trained on the original scale throughout. This convention determines the interpretation of the Elastic Net odds ratios reported in §2.13 Model Interpretability.

### 2.10 Threshold selection

Classification thresholds were determined using development-set cross-validated out-of-fold predictions (under time-block CV), rather than apparent predictions from the final fitted model, to reduce optimistic bias in threshold selection. The Youden index (maximizing sensitivity + specificity − 1) was used as a prespecified statistical operating point for descriptive reporting of classification performance. It was retained as a widely used reporting convention that facilitates comparison with other prediction studies. It was not defined as a treatment threshold because it assigns equal weight to sensitivity and specificity and does not incorporate the unequal clinical consequences of delayed effective therapy and unnecessary anti-VRE exposure. Additional high-specificity (≥ 0.95) and high-negative-predictive-value (NPV ≥ 0.95) operating points were calculated as supplementary descriptive analyses.

### 2.11 Model evaluation

All models were evaluated once in the temporally held-out validation set. Discrimination was assessed using the area under the receiver operating characteristic curve (AUROC; **Figure 2A**) and the area under the precision-recall curve (AUPRC; **Supplementary Figure S3**). Overall prediction error was assessed using the Brier score. At the Youden index threshold, sensitivity, specificity, positive predictive value (PPV), and negative predictive value (NPV) were computed for each model and tabulated alongside the discrimination metrics (**Figure 2B**). These classification metrics describe model behavior at a common statistical operating point and were not used to recommend treatment decisions. Bootstrap 95% confidence intervals (CIs) for AUROC, AUPRC, and the Brier score were estimated from 1,000 paired resamples of the validation set with a fixed random seed, so that the CIs are directly comparable across models.

**Figure 2:**
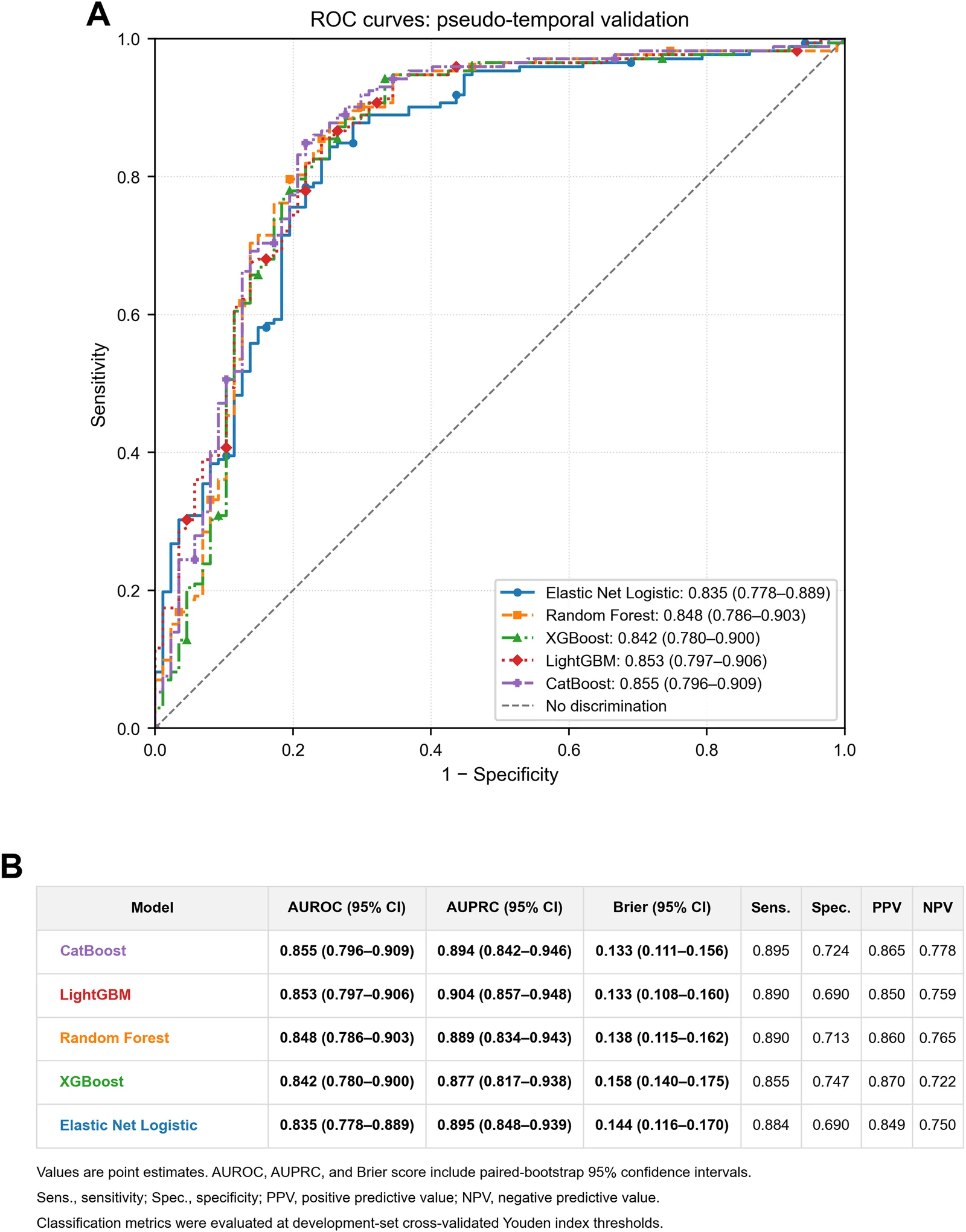
Discrimination of the five candidate models on the pseudo-temporal validation set. **(A)** Receiver operating characteristic (ROC) curves for the Elastic Net logistic regression reference model and the four tree-based comparators (Random Forest, XGBoost, LightGBM, and CatBoost) on the pseudo-temporal validation set (n = 259, of whom 172 were VRE). Models are distinguished by color, line type, and plotting symbol to remain identifiable in grayscale and for readers with color-vision deficiency. The legend reports each model’s area under the ROC curve (AUROC) with the 95% bootstrap confidence interval derived from 1,000 paired resamples of the validation set using a fixed random seed. The diagonal dashed line indicates the no-discrimination reference (AUROC = 0.50). **(B)** Summary performance metrics for the same five models on the pseudo-temporal validation set. For each model, the table reports the AUROC, area under the precision-recall curve (AUPRC), and Brier score, each with its 95% bootstrap confidence interval (1,000 paired resamples; identical resampling indices across models so that the confidence intervals are directly comparable), as well as the sensitivity, specificity, positive predictive value (PPV), and negative predictive value (NPV) at the Youden index threshold derived from the corresponding development-set cross-validated out-of-fold predictions. This operating point was used for descriptive classification reporting and should not be interpreted as a recommended treatment threshold. CatBoost achieved the highest AUROC, LightGBM achieved the highest AUPRC, and CatBoost and LightGBM had the same Brier score after rounding to three decimal places. No pairwise difference in AUROC reached statistical significance after Bonferroni or Benjamini–Hochberg correction across the 10 model pairs (**Supplementary Table S7**). AUPRC, area under the precision-recall curve; AUROC, area under the receiver operating characteristic curve; CI, confidence interval; NPV, negative predictive value; PPV, positive predictive value.

### 2.12 Calibration assessment

Calibration was evaluated using four complementary metrics. (i) The *calibration slope* and *calibration intercept* were estimated by regressing the binary outcome on the logit of predicted probabilities; a slope of 1.0 and an intercept of 0.0 indicate perfect calibration. (ii) *Calibration-in-the-large* (CITL) was estimated as the intercept of a binomial generalized linear model in which the logit of the predicted risk was entered as an offset; CITL captures average over- or under-prediction. (iii) The *expected calibration error* (ECE) was computed using 10 equal-width bins of predicted probability. (iv) *Lowess-smoothed calibration curves* visualized the relationship between predicted and observed VRE frequency (**Figure 3A**). Bootstrap 95% CIs for the calibration slope and CITL were computed from 1,000 resamples of the validation set.

**Figure 3:**
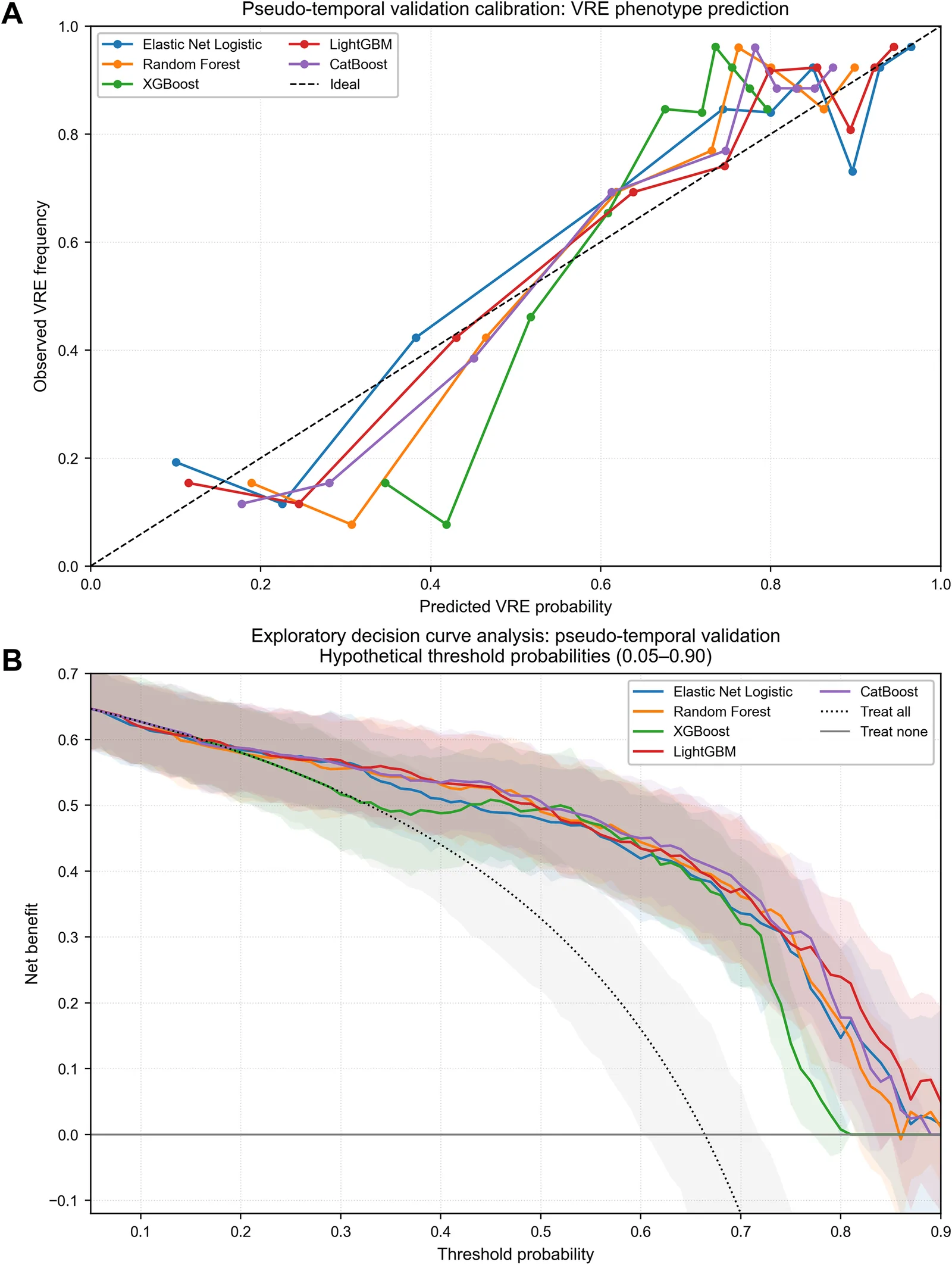
Calibration and exploratory decision curve analysis of the five candidate models on the pseudo-temporal validation set. **(A)** Quantile-binned calibration curves comparing predicted probabilities of VRE (x-axis) with observed VRE frequency (y-axis) on the pseudo-temporal validation set (n = 259). The diagonal dashed line indicates perfect calibration, and symbols represent observed event frequencies within 10 quantile-based bins. **Supplementary Table S9** reports each model’s calibration slope, calibration intercept, calibration-in-the-large (CITL), expected calibration error (ECE), and bootstrap 95% CIs for slope and CITL. Elastic Net and LightGBM showed the closest agreement between predicted and observed risk (slopes, 0.91 and 1.08, respectively); XGBoost showed marked under-dispersion (slope, 2.41). Models are distinguished by color and plotting symbol. **(B)** Exploratory decision curve analysis showing model-based net benefit across a prespecified range of hypothetical threshold probabilities (0.05–0.90). The treat-all strategy (dotted black line) and treat-none strategy (solid grey line at zero net benefit) are mathematical references rather than observed usual-care strategies. Thresholds were not elicited from clinicians or intended as treatment recommendations. Paired bootstrap 95% confidence bands were calculated from 1,000 validation-set resamples and are shown for the five model curves and the treat-all reference. CITL, calibration-in-the-large; DCA, decision curve analysis; ECE, expected calibration error.

For the Elastic Net model, exploratory logistic recalibration (fitting an intercept and slope on the validation-set predictions) was to be performed only if its calibration slope fell outside an analysis-defined range of 0.90–1.10. This narrow range was chosen as a pragmatic tolerance around the ideal value of 1.0, rather than as a universally validated clinical acceptability threshold. The trigger was not preregistered or specified in a study protocol before validation-set results were examined and therefore should not be interpreted as a prospectively prespecified criterion. Had the trigger fired, recalibration parameters fitted to the pseudo-temporal validation outcomes would have been reported only as *post hoc* descriptive adjustments; primary discrimination and calibration metrics would not have been re-estimated on those recalibrated predictions or presented as evidence of generalizable improvement.

### 2.13 Model comparison

Pairwise differences in AUROC between the five models were assessed using paired DeLong tests on the pseudo-temporal validation predictions. Both Bonferroni and Benjamini–Hochberg corrections were applied across the 10 model pairs. With only 259 validation admissions, non-significant results cannot rule out small but clinically meaningful differences and were interpreted as absence of evidence rather than equivalence.

### 2.14 Decision curve analysis

Decision curve analysis (DCA) was performed as an exploratory analysis to estimate net benefit across threshold probabilities representing different relative weights assigned to false-positive and false-negative classifications. The threshold-probability range of 0.05–0.90 was selected to examine a broad set of hypothetical risk tolerances; these thresholds were not elicited from clinicians, linked to a prespecified treatment policy, or intended as treatment recommendations. Model curves were compared with the mathematical reference strategies of treating all patients and treating none (**Figure 3B**), which should not be interpreted as observed usual-care strategies. Uncertainty was quantified with 1,000 paired validation-set bootstrap resamples at thresholds 0.05–0.90 in increments of 0.01. Each resample recomputed model, treat-all, and treat-none net benefit. Paired model-minus-treat-all differences were prespecified for thresholds 0.10, 0.20, 0.30, and 0.50.

### 2.15 Model interpretability

For the Elastic Net reference model, coefficients were transformed into odds ratios. Because continuous predictors had been standardized to zero mean and unit variance within the development-set pipeline (see §2.9 Model Development), the odds ratio for a continuous predictor corresponds to a one-standard-deviation increase. For ordinary binary predictors retained on their original 0/1 scale, the odds ratio corresponds to presence versus absence. For retained indicators derived from a categorical variable, the odds ratio compares that level with the source variable’s composite reference group defined in §2.6 Missing Data and Preprocessing: *E. faecium* and *E. faecalis* were each compared with the combined other-species/species-not-specified group, whereas peritoneal fluid and tissue were each compared with the group comprising all other specimen-source levels. These are conditional model-coefficient contrasts, with the other retained predictors held fixed. Bootstrap 95% CIs for the odds ratios were obtained from 1,000 resamples of the development set. The odds ratios and CIs were used for descriptive interpretation of the fitted predictive model; because they followed data-driven feature selection and penalized estimation, they were not intended for confirmatory inference about independent risk factors or causal effects. **Figure 4A** displays the corresponding model-entry-scale coefficients on the log-odds scale; bars are ordered by absolute coefficient magnitude.

**Figure 4:**
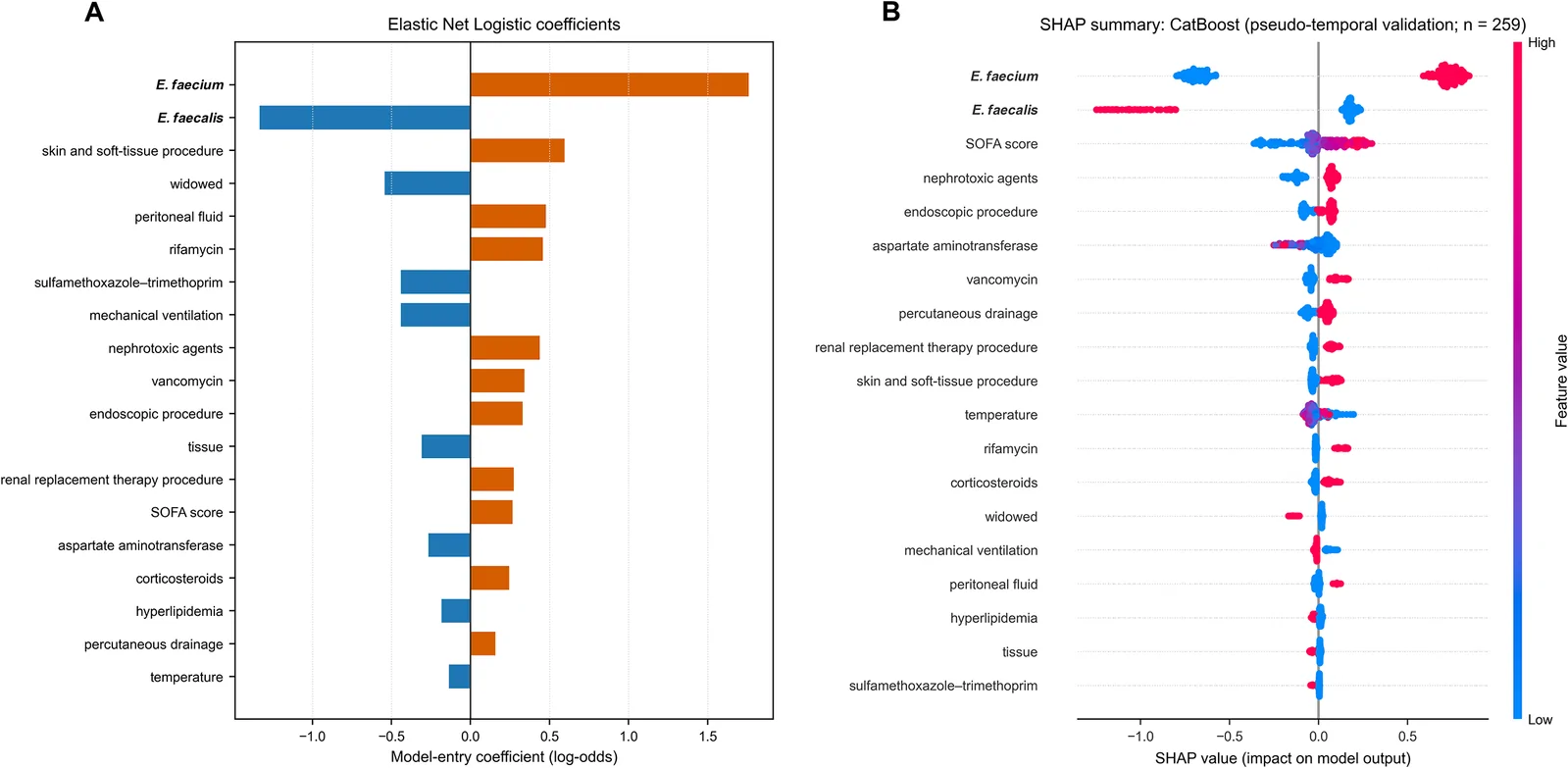
Variable importance for the Elastic Net reference model and the CatBoost comparator. **(A)** Model-entry-scale coefficients of the 19 final predictors retained in the Elastic Net logistic regression model fitted to the full development set (n = 552). Before final selection, binary and one-hot encoded predictors were required to have at least 20 exposed observations and at least 5 observations in every predictor-by-outcome cell in the development set. Continuous predictors were standardized to zero mean and unit variance within the development-set pipeline, so their coefficients represent the log-odds change per one-standard-deviation increase. Ordinary binary predictors were retained on their original 0/1 scale and represent presence versus absence. Retained categorical indicators represent contrasts with their source variable’s composite reference group: other *Enterococcus* species/species not specified for the two species indicators, and all specimen sources other than peritoneal fluid and tissue for the two specimen-source indicators. Bars are ordered by absolute coefficient magnitude; positive values are shown in orange and negative values in blue. Odds ratios with 95% bootstrap confidence intervals (1,000 resamples), bootstrap selection frequencies, and reference-group definitions are reported in **Supplementary Table S6**. These post-selection quantities describe the fitted predictive model and are not estimates of independent risk-factor or causal effects. **(B)** SHAP (SHapley Additive exPlanations) beeswarm summary plot for the CatBoost model in all 259 pseudo-temporal-validation admissions. Each dot represents one validation-set observation; horizontal position indicates the SHAP value (the contribution of that feature to the model’s log-odds output for that observation), with positive values shifting the prediction toward VRE and negative values shifting it away from VRE. Color encodes the underlying feature value (blue, low; red, high). Features are ordered from top to bottom by mean absolute SHAP value in the full validation set; the corresponding numerical rankings are tabulated in **Supplementary Table S11**. *E. faecium* and *E. faecalis* dominate the global importance ranking, followed by SOFA score and exposure to nephrotoxic agents. SHAP, SHapley Additive exPlanations; SOFA, Sequential Organ Failure Assessment.

For CatBoost—identified as the best-performing model by pseudo-temporal-validation AUROC—TreeSHAP was used as a *post hoc* interpretability analysis. Mean absolute SHAP values were computed for all 259 pseudo-temporal-validation admissions to rank features, and a SHAP beeswarm summary plot was generated from the full validation set to visualize both the magnitude and the direction of feature contributions (**Figure 4B**). Two illustrative SHAP waterfall plots—one true-negative low-risk case and one true-positive high-risk case—were selected *post hoc* from the full validation set to illustrate how individual predictions are constructed from feature contributions (**Figures 5A, B**); these examples are illustrative and do not represent population-average model behavior. The complete Elastic Net scoring specification (intercept, coefficients, and development-set standardization parameters) is provided in **Supplementary Table S16** to enable independent evaluation.

**Figure 5:**
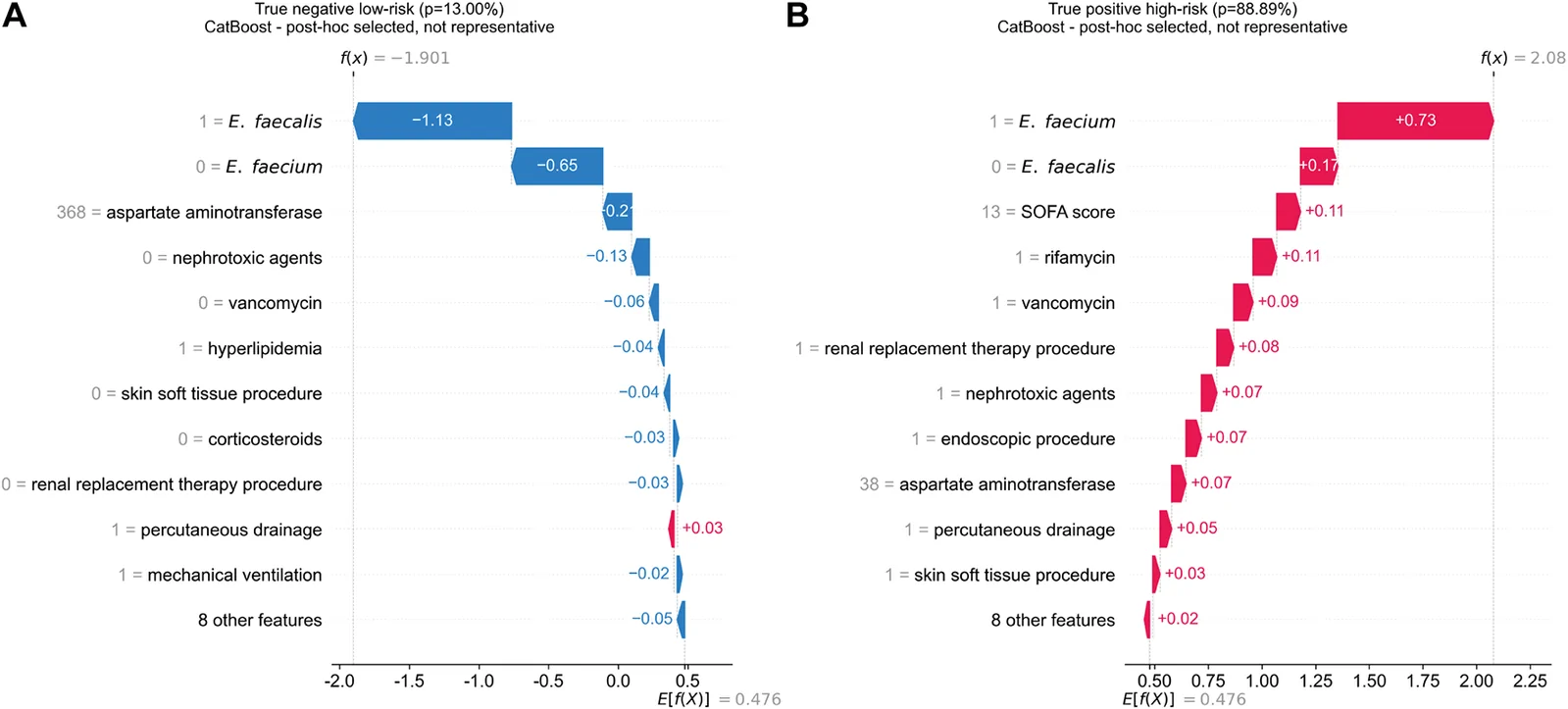
Illustrative SHAP waterfall plots for two individual CatBoost predictions on the pseudo-temporal validation set. Both panels show the additive feature contributions (SHAP values) that move the CatBoost model output from the baseline TreeSHAP expected value of the development-set-fitted model, *E*[*f*(*X*)] = 0.476, to the case-specific log-odds, *f*(*x*). The corresponding predicted VRE probability is reported in each panel title. Positive SHAP values (red) push the prediction toward VRE; negative SHAP values (blue) push it away from VRE. Features with the smallest absolute contributions are aggregated into the “other features” row at the bottom. **(A)** True-negative low-risk example (predicted probability of VRE, 13.0%). The downward shift was driven primarily by the presence of *E. faecalis* (SHAP, −1.13), the absence of *E. faecium* (−0.65), and elevated aspartate aminotransferase (−0.21). **(B)** True-positive high-risk example (predicted probability of VRE, 88.9%). The largest upward contributions were the presence of *E. faecium* (+0.73), the absence of *E. faecalis* (+0.17), a SOFA score of 13 (+0.11), rifamycin exposure (+0.11), and vancomycin exposure (+0.09). Both cases were selected *post hoc* and are presented for illustrative purposes only; they do not represent population-average model behavior. SHAP, SHapley Additive exPlanations; SOFA, Sequential Organ Failure Assessment.

### 2.16 Internal validation and optimism correction

Optimism in apparent AUROC was estimated by bootstrap resampling (200 resamples of the development set) with fixed hyperparameters selected from the original development-set cross-validation. For each bootstrap sample, the model was refitted; optimism was estimated as the difference between the AUROC on the bootstrap sample (apparent performance) and the AUROC of the bootstrap-fitted model applied to the original full development set. The mean optimism across the 200 resamples was subtracted from the apparent AUROC of the final model fitted on the full development set to obtain the optimism-corrected AUROC, which represents an internally validated estimate on the development set and is distinct from the pseudo-temporal-validation AUROC. Because hyperparameters were not re-tuned within each bootstrap iteration, this procedure captures optimism from data-driven coefficient estimation but does not capture optimism from hyperparameter selection; the reported optimism should therefore be interpreted as a lower bound of the total internal-validation optimism. The number of resamples (200) was selected as a computational compromise; conventional recommendations for optimism-correction bootstrap typically use ≥ 200 resamples, but greater numbers can further stabilize the optimism-SD estimate.

### 2.17 Sensitivity analyses

Missing data and imputation. The distribution of missing values across predictors in the development and validation sets was summarized. Sensitivity analyses used the same 19 final predictors and the fixed final Elastic Net architecture (*C* = 0.281177, *l1_ratio* = 0.1; SAGA solver; continuous predictors standardized using development-set parameters). The primary candidate-level preprocessing pipeline—mode imputation for binary predictors, single iterative imputation for continuous predictors, and one-hot encoding for categorical predictors—was compared with a pipeline in which continuous predictors were median-imputed. Imputation and scaling were fitted in the development set and applied to the pseudo-temporal validation set. A complete-case analysis using the same 19-predictor Elastic Net architecture without imputation was also performed after excluding admissions with a missing value in any final predictor. Because complete-case restriction changes both sample size and the composition of the analyzed population, differences from the full-sample analyses were not attributed to sample-size reduction alone. In an additional multiple-imputation sensitivity analysis, 10 posterior-draw iterative imputations were generated, the fixed 19-predictor Elastic Net was refitted in each imputed development dataset, and performance was evaluated in its corresponding imputed validation dataset. Within-imputation variances for AUROC and AUPRC were estimated from 200 bootstrap resamples of the held-out validation predictions; logit-transformed performance estimates were combined with between-imputation variance using Rubin’s rules and back-transformed for reporting. Performance after averaging predicted probabilities across imputations was also reported.

Descriptive comparisons. Continuous variables were compared between two groups using the Wilcoxon rank-sum test. For binary predictors and for retained one-hot encoded indicators derived from multilevel categorical variables (species, specimen source), Fisher’s exact test was used to compare presence versus absence of each indicator; no single omnibus *P* value for the underlying source variable is reported. Standardized mean differences (SMDs) were emphasized for describing the magnitude of between-group differences.

Demographic subgroup performance. Model performance (AUROC, AUPRC) was evaluated descriptively within subgroups defined by age (< 65 vs. ≥ 65 years) and sex (female vs. male). No formal interaction tests were performed.

Prevalence-adjusted predictive values. As an exploratory mathematical analysis, sensitivity and specificity of the Elastic Net model at the Youden index threshold were held fixed while PPV and NPV were recalculated for assumed VRE prevalences ranging from 0.05 to 0.70 using Bayes’ theorem. This analysis was not used to select or recommend a treatment threshold.

Multicollinearity. Variance inflation factors (VIFs) were computed for the 19 final predictors in the development set. A Spearman rank correlation matrix was generated as a complementary check (**Supplementary Figure S5**).

Reviewer-requested sensitivity analyses. All refitting and imputation used development data only, with random seed 42; the pseudo-temporal validation set was reserved for final evaluation. We compared the original 19-predictor Elastic Net with a 17-predictor model excluding both species indicators, using a paired DeLong test. We forced standardized time from ICU admission to culture into a 20-predictor model and bootstrapped coefficients for six exposure/procedure variables. Platt and isotonic recalibrators were fitted separately for all five models using development-set out-of-fold predictions and then applied unchanged to validation predictions. Race/ethnicity performance was descriptive: recorded White versus recorded non-White was the main comparison, with recorded Black, Hispanic, Asian, and other/unknown groups displayed with small-sample caveats.

The original coefficient-only optimism analysis was supplemented by a 100-resample selection-inclusive bootstrap. Within each outer resample, development-support screening, 100 fixed-hyperparameter stability-selection resamples, the prespecified 20-variable cap, and final fitting were repeated. Because preprocessing and hyperparameters were fixed, this was not described as a complete repetition of every tuning decision. Aspartate aminotransferase was removed in an 18-predictor sensitivity model; its coefficient direction was also assessed under iterative, median, and 10 posterior-draw imputations. Finally, Elastic Net and LightGBM performance was summarized descriptively within anchor-year groups labelled 2017–2019 and 2020–2022. The analysis-ready data do not contain excluded cultures without vancomycin AST and do not retain ICU stay_id or ICU timestamps; quantitative comparison of AST-missing isolates and re-audit of culture attribution across multiple ICU stays were therefore not possible.

### 2.18 Software

All analyses were conducted in Python 3.11.5, using scikit-learn 1.3.1, XGBoost 3.1.1, LightGBM 4.6.0, CatBoost 1.2.8, statsmodels 0.14.5, SHAP 0.49.1, pandas 2.3.1, numpy 1.26.4, matplotlib 3.10.0, and seaborn 0.13.2. The analysis configuration and the random seed were archived with the pipeline outputs.

### 2.19 Data and code availability

The MIMIC-IV database is publicly available at https://physionet.org/content/mimiciv/ following completion of required training and credentialing. The public analysis repository is available at https://github.com/changyanbin1993/VRE-ICU-prediction. The analysis programs were updated for this revision and include the complete revision pipeline, the fitted model and preprocessing objects, the environment file, and a model-object manifest. The external validation study has not started and is future work.

## 3 Results

### 3.1 Study population and baseline characteristics

Across the MIMIC-IV anchor-year groups labelled 2008–2022, 811 ICU admissions with a sterile-site *Enterococcus*-positive culture obtained more than 48 hours after ICU admission were identified (**Figure 1**). After selecting the first eligible culture per ICU admission as the index culture, 507 ICU admissions (62.5%) were classified as vancomycin-resistant *Enterococcus* (VRE) and 304 (37.5%) as vancomycin-susceptible *Enterococcus* (VSE). The cohort was split pseudo-temporally by anchor-year groups labelled 2008–2016 for development (n = 552; VRE prevalence, 60.7%) and 2017–2022 for pseudo-temporal validation (n = 259; VRE prevalence, 66.4%) (**Figure 1**; **Table 1**; **Supplementary Table S3**).

The distributions of the 19 final model predictors in the full cohort are summarized by vancomycin-resistance outcome in **Table 1**. *E. faecium* accounted for 73.4% of VRE isolates but only 18.4% of VSE isolates (standardized mean difference [SMD], 1.32), whereas *E. faecalis* accounted for 37.2% of VSE isolates but only 3.0% of VRE isolates (SMD, −0.95). Tissue cultures were more frequent in the VSE group (25.3% vs. 12.8%; SMD, −0.32), while peritoneal-fluid cultures had similar frequencies in the two groups (13.2% vs. 13.8%; SMD, 0.02).

Among the displayed final clinical predictors (**Table 1**), VRE cases had higher SOFA scores (median 9, IQR 6–13 vs. 7, IQR 4–11; SMD, 0.41), a higher rate of nephrotoxic agent exposure (71.8% vs. 50.3%; SMD, 0.45), and higher exposures to several antimicrobials, including vancomycin (33.1% vs. 22.4%; SMD, 0.24) and rifamycin (16.2% vs. 4.9%; SMD, 0.37). Endoscopic procedures (59.4% vs. 46.7%; SMD, 0.26) and renal replacement therapy (37.3% vs. 23.0%; SMD, 0.31) were also more common in the VRE group, whereas mechanical ventilation was slightly less common (77.5% vs. 84.5%; SMD, −0.18). Time from ICU admission to specimen collection was longer in the VRE group than in the VSE group in the overall cohort (median 363 hours, IQR 216–626, vs. 208 hours, IQR 104–469; SMD, 0.34), consistent with later ICU-acquired or ICU-persistent culture-positive episodes in many VRE cases. This variable was evaluated as a candidate predictor but did not meet the bootstrap stability threshold in feature selection (selection frequency < 0.50) and was not retained in the final 19-predictor set. Its lower selection frequency may reflect correlation with retained predictors that partially overlap with time-dependent care intensity, but this was not formally quantified. In the development-versus-validation comparison, most SMDs were below 0.2, although several comorbidity and procedure variables showed larger temporal differences (**Supplementary Table S3**). The observed increase in VRE prevalence from 60.7% in the development set to 66.4% in the pseudo-temporal validation set was consistent with the trend toward higher VRE rates in later anchor-year groups (**Supplementary Figure S1**).

### 3.2 Feature selection

Of 141 candidate processed predictors, Elastic Net logistic regression with time-block cross-validation within the development set identified 34 with non-zero coefficients. Bootstrap stability analysis (100 resamples) identified 28 predictors meeting the stability threshold (selection frequency ≥ 0.50); their frequencies are visualized in **Supplementary Figure S2**. Development-set support screening excluded nine stable binary predictors that did not satisfy the prespecified minimum of 20 exposed observations and 5 observations in every predictor-by-outcome cell: linezolid, foreign body, mupirocin, head trauma, biopsy, dialysis fluid, silver sulfadiazine, macrolide, and aminoglycoside (**Supplementary Table S5**). Nineteen predictors met both the stability and support criteria and were retained, below the prespecified maximum of 20. With VRE defined as the event, the conventional EPV was 17.63 (335 VRE events/19 predictors); the minimum outcome-class observations per predictor was 11.42 (217 VSE observations/19 predictors). The retained predictors spanned *Enterococcus* species, specimen source, severity, antimicrobial exposures, procedures, comorbidity, laboratory values, and temperature (**Supplementary Table S4**).

### 3.3 Model performance

The five models—Elastic Net logistic regression (the interpretable reference), Random Forest, XGBoost, LightGBM, and CatBoost—were trained on the same 19 predictors selected by Elastic Net and evaluated once in the pseudo-temporal validation set. Discrimination was comparable across models (**Figures 2A, B**; **Supplementary Figure S4**). CatBoost achieved the highest pseudo-temporal-validation AUROC (0.855; 95% bootstrap CI, 0.796–0.909), followed by LightGBM (0.853; 95% CI, 0.797–0.906), Random Forest (0.848; 95% CI, 0.786–0.903), XGBoost (0.842; 95% CI, 0.780–0.900), and Elastic Net (0.835; 95% CI, 0.778–0.889). AUPRC values ranged from 0.877 (XGBoost) to 0.904 (LightGBM), all substantially exceeding the validation-set prevalence baseline of 0.664 (**Supplementary Figure S3**). Brier scores ranged from 0.133 (CatBoost and LightGBM, tied to three decimal places) to 0.158 (XGBoost); the complete Brier scores with 95% bootstrap CIs for all five models are reported in **Figure 2B**. Pairwise DeLong tests showed no statistically significant difference in AUROC between any model pair after Bonferroni correction (all adjusted *P* > 0.05; **Supplementary Table S7**). Within this common Elastic-Net-selected predictor set, the comparisons did not demonstrate statistically superior discrimination for any model, although they were not equivalence tests.

At the prespecified Youden index threshold derived from development-set cross-validated predictions, sensitivity ranged from 0.855 to 0.895 and specificity from 0.690 to 0.747 across the five models in the pseudo-temporal validation set (**Figure 2B**). The Elastic Net reference model correctly classified 152 of 172 VRE cases (sensitivity, 0.884) and 60 of 87 VSE cases (specificity, 0.690), corresponding to a PPV of 0.849 and an NPV of 0.750. Among the comparators, CatBoost achieved a sensitivity of 0.895, specificity 0.724, PPV 0.865, and NPV 0.778; LightGBM, 0.890, 0.690, 0.850, and 0.759; Random Forest, 0.890, 0.713, 0.860, and 0.765; and XGBoost, 0.855, 0.747, 0.870, and 0.722, respectively. These results describe classification performance at a statistical operating point rather than a recommended treatment threshold. Classification metrics at supplementary high-specificity and high-NPV operating points are reported in **Supplementary Table S10**.

Bootstrap optimism correction (200 resamples; fixed hyperparameters), applied to the development-set apparent AUROC, estimated mean optimism ranging from 0.019 (Elastic Net) to 0.055 (XGBoost); the resulting optimism-corrected AUROCs were 0.851 to 0.869 (**Supplementary Table S8**). These development-set estimates remained higher than the corresponding pseudo-temporal-validation AUROCs (0.835–0.855); for XGBoost, the optimism-corrected estimate was 0.869 versus 0.842 in pseudo-temporal validation. The residual attenuation may reflect the joint effects of distributional drift and development-period overfitting or model-selection optimism not removed by the simplified fixed-hyperparameter bootstrap. The rank order of the optimism-corrected AUROCs did not exactly match the pseudo-temporal-validation ranking; these estimates capture different sources of uncertainty, and small point-estimate differences do not support a stable model ranking.

### 3.4 Calibration

Calibration slopes, calibration intercepts, CITL values, and ECE values for all five models are reported in **Supplementary Table S9**, and the calibration curves are visualized in **Figure 3A**. Slopes closest to 1.0 were observed for Elastic Net (0.91; 95% CI, 0.71–1.18) and LightGBM (1.08; 0.83–1.41). Elastic Net had a CITL of 0.10 (95% CI, −0.22 to 0.43) and an ECE of 0.076. Random Forest (slope, 1.41), CatBoost (1.46), and particularly XGBoost (2.41) showed under-dispersion (calibration slope > 1, indicating predicted probabilities clustered toward the mean rather than spanning the full 0-to-1 range appropriately). The slope confidence intervals included 1 for Elastic Net and LightGBM but excluded 1 for the other three models. Thus, despite discrimination close to the best-performing comparators, the uncalibrated XGBoost, random-forest, and CatBoost probabilities require recalibration before being interpreted as absolute risks for threshold-based decisions. Because the Elastic Net slope was within the analysis-defined 0.90–1.10 range, exploratory recalibration was not performed. This range was not prospectively prespecified, as noted in the Methods. These findings support local calibration assessment before probability-based deployment at a new site.

### 3.5 Decision curve analysis

In the exploratory decision curve analysis, the model curves were above the treat-none reference at evaluated thresholds below approximately 0.85 and above the treat-all reference from approximately 0.21 to the upper end of the evaluated range (**Figure 3B**). The curves for the five models were closely clustered. For the Elastic Net model, net benefit was 0.564 at a threshold probability of 0.30 and 0.479 at 0.50; at 0.30, the difference from the treat-all reference was 0.044. These are descriptive, model-based estimates under the assumptions of DCA. The threshold probabilities were hypothetical, treat-all and treat-none were mathematical references rather than observed care strategies, and paired bootstrap 95% confidence bands are shown for every curve and were calculated from 1,000 resamples.

### 3.6 Variable importance and model interpretability

In the Elastic Net model, binary and one-hot encoded predictors were retained on their original 0/1 scale, whereas SOFA score, aspartate aminotransferase, and temperature were standardized. Final-model tuning selected *C* = 0.281, which was not at a search boundary, and *l1_ratio* = 0.1, the lower bound of the searched grid. Relative to the composite reference group of other *Enterococcus* species and *Enterococcus* spp. without species specification, *E. faecium* was associated with higher predicted odds (OR, 5.81; 95% bootstrap CI, 4.13–8.61; bootstrap selection frequency, 1.00), whereas *E. faecalis* was associated with lower predicted odds (OR, 0.26; 95% CI, 0.17–0.41; selection frequency, 1.00) (**Figure 4A**, **Supplementary Table S6**). Odds ratios for ordinary binary predictors represent presence versus absence, those for retained categorical indicators represent the contrast with the corresponding composite reference group, and those for continuous predictors are per one-standard-deviation increase. Other positive coefficients included skin and soft tissue procedure (OR, 1.81; 95% CI, 1.21–2.71), peritoneal fluid versus all other specimen sources except tissue (OR, 1.61; 1.07–2.46), rifamycin (OR, 1.58; 1.03–2.43), nephrotoxic agents (OR, 1.55; 1.06–2.29), and SOFA score (OR, 1.31; 1.07–1.61); aspartate aminotransferase (OR, 0.77; 0.61–0.95) and mechanical ventilation (OR, 0.64; 0.41–1.00) had negative coefficients. The full list and reference-group definitions are provided in **Supplementary Table S6**. These post-selection penalized estimates describe the fitted predictive model and should not be interpreted as confirmatory independent risk-factor or causal effects. Variance inflation factors were below 2.3 for all 19 predictors (**Supplementary Table S14**), indicating acceptable multicollinearity; the Spearman correlation matrix (**Supplementary Figure S5**) confirmed that pairwise correlations were generally weak to moderate.

In the CatBoost model (the best-performing comparator by pseudo-temporal-validation AUROC), SHAP analysis in all 259 pseudo-temporal-validation admissions ranked *E. faecium* (mean |SHAP|, 0.715), *E. faecalis* (0.324), SOFA score (0.114), and nephrotoxic agents (0.094) as the four most important features (**Figure 4B**; **Supplementary Table S11**). Directional patterns were broadly consistent with the Elastic Net coefficients for the leading predictors: *E. faecium*, higher SOFA scores, and nephrotoxic-agent exposure shifted predictions toward VRE, whereas *E. faecalis* and higher aspartate aminotransferase shifted predictions away from VRE. Differences in lower-ranked features were expected because the linear and tree-based models capture predictor effects differently.

Two illustrative SHAP waterfall plots are presented as case studies of how individual CatBoost predictions are constructed from feature contributions (**Figures 5A, B**). In a true-negative low-risk example (**Figure 5A**; predicted probability, 13.0%), the prediction was driven downward primarily by *E. faecalis* being present (SHAP, −1.13), *E. faecium* being absent (−0.65), and elevated aspartate aminotransferase (−0.21). In a true-positive high-risk example (**Figure 5B**; predicted probability, 88.9%), the largest upward contributions were *E. faecium* being present (+0.73), *E. faecalis* being absent (+0.17), a SOFA score of 13 (+0.11), rifamycin exposure (+0.11), and vancomycin exposure (+0.09). These examples were selected *post hoc* and are illustrative; they do not represent population-average model behavior.

### 3.7 Sensitivity analyses

#### 3.7.1 Missing data and imputation strategy

Four laboratory variables had missingness exceeding 25% in the development set: total bilirubin (28.3%), alanine aminotransferase (28.3%), aspartate aminotransferase (27.9%), and lactate (26.6%) (**Supplementary Table S1**). All other predictors had less than 5% missing data. In sensitivity analyses using the same 19 final predictors and fixed Elastic Net architecture (*C* = 0.281177, *l1_ratio* = 0.1), replacing single iterative imputation with median imputation changed the pseudo-temporal-validation AUROC by 0.0007 (0.8348 vs. 0.8341) and the AUPRC by 0.0001 (0.8945 vs. 0.8944; **Supplementary Table S2**). Across 10 posterior-draw imputations, the Rubin-rules pooled AUROC was 0.8393 (95% CI, 0.7761–0.8873) and the pooled AUPRC was 0.8971 (0.8410–0.9349); averaging the imputation-specific predicted probabilities yielded an AUROC of 0.8406 and an AUPRC of 0.8983. Complete-case analysis retained 386 development admissions and 206 validation admissions and yielded an AUROC of 0.8166 and an AUPRC of 0.9027. The lower complete-case AUROC may reflect reduced sample size, selection into the complete-case subset, and changes in case mix; the analysis does not isolate the contribution of any one mechanism. These results support stability of discrimination across single and multiple-imputation strategies.

#### 3.7.2 Subgroup performance

Demographic subgroup analyses (age < 65 vs. ≥ 65 years; female vs. male) showed that model performance was broadly consistent across strata (**Supplementary Table S12**). For Elastic Net, the AUROC ranged from 0.808 (age ≥ 65) to 0.873 (female); across all five models and four subgroups, the maximum spread was 0.077 (CatBoost in females, 0.885, vs. Elastic Net in patients aged ≥ 65, 0.808). These are descriptive subgroup results; no formal interaction tests were performed, and the modest subgroup sample sizes (range, 101–158) limit the precision of these estimates.

#### 3.7.3 Prevalence-adjusted predictive values

Holding the Elastic Net model’s sensitivity (0.884) and specificity (0.690) at the Youden index threshold fixed, PPV and NPV were recalculated across assumed VRE prevalences from 0.05 to 0.70 (**Supplementary Table S13**). At an assumed prevalence of 0.10, the calculated PPV was 0.240 and the NPV was 0.982; at the overall cohort VRE prevalence of 0.625 (507/811), the corresponding values were 0.826 and 0.781. These estimates are mathematical projections under fixed operating characteristics and do not establish rule-in, rule-out, or treatment performance in settings with different prevalence.

#### 3.7.4 Species-level identification

Removing both species indicators—exactly the genus-level-information scenario because species information was carried only by these two indicators—reduced AUROC from 0.835 to 0.657 (95% CI, 0.588–0.732), AUPRC from 0.895 to 0.781, and calibration slope from 0.91 to 0.57 (paired DeLong *P* < 0.001; **Supplementary Table S19**). This complementary-information ablation directly quantifies the incremental predictive contribution of timely species-level identification and the performance expected when only genus-level information is available.

#### 3.7.5 Adjustment for time to culture

Forcing standardized time to culture into the fixed model changed AUROC from 0.835 to 0.836 (paired DeLong *P* = 0.364) and AUPRC from 0.895 to 0.897 (**Supplementary Table S20**). Its coefficient was small (OR per SD, 1.08; bootstrap 95% CI, 0.87–1.51). The largest absolute coefficient change among the six reviewer-specified exposure/procedure variables was 8.9% for endoscopic procedure; corresponding changes were 0.9% for nephrotoxic agents, −2.2% for rifamycin, −7.7% for corticosteroids, −4.5% for renal replacement therapy procedure, and −1.1% for percutaneous drainage. Thus, this adjustment did not materially alter predictive performance or these fitted associations.

#### 3.7.6 Recalibration

Recalibrators were fitted only to development-set OOF predictions (**Supplementary Table S21**; **Supplementary Figure S6**). Platt scaling changed validation slopes for Random Forest, XGBoost, and CatBoost from 1.41, 2.41, and 1.46 to 0.94, 1.02, and 1.01; their Brier scores improved from 0.138, 0.158, and 0.133 to 0.131, 0.132, and 0.126, respectively. Isotonic regression further reduced ECE but overcorrected the slopes of Random Forest (0.48) and XGBoost (0.41); CatBoost remained close to ideal (1.00). These findings favor Platt scaling as the more stable simple recalibration option in these data and make recalibration a prerequisite for probability-based use of the three originally under-dispersed tree models.

#### 3.7.7 Recorded race/ethnicity

Elastic Net AUROC was 0.819 among recorded White admissions (n = 151; 97 events) and 0.864 among recorded non-White admissions (n = 108; 75 events) in pseudo-temporal validation (**Supplementary Table S22**). Recorded Black (n = 17), Hispanic (n = 13), and Asian (n = 15) groups were too small for reliable performance estimates; their values and the other/unknown group (n = 63) are displayed only for completeness. These descriptive results neither establish fairness nor demonstrate equivalence.

#### 3.7.8 Selection-inclusive optimism

The selection-inclusive bootstrap repeated support screening, 100 stability-selection resamples, feature capping, and final fitting in each of 100 outer resamples while keeping preprocessing and hyperparameters fixed (**Supplementary Table S23**). All 100 outer resamples succeeded. Mean optimism was 0.040 (95% empirical interval, 0.010–0.067), and the median number of selected predictors was 17 (IQR, 15–19). Results are reported as a computational sensitivity analysis rather than a complete repetition of all tuning steps.

#### 3.7.9 Aspartate aminotransferase

No missingness indicator was retained among the final 19 predictors. Removing aspartate aminotransferase increased validation AUROC from 0.835 to 0.845 (paired DeLong *P* = 0.099) and produced a calibration slope of 0.99 (**Supplementary Table S24**). The coefficient remained negative with iterative imputation (OR, 0.75), median imputation (0.76), and all 10 posterior-draw imputations (OR range, 0.76–0.92). This directional stability does not support a causal or protective interpretation.

#### 3.7.10 Decision-curve uncertainty and anchor-year-group trends

At thresholds 0.10 and 0.20, Elastic Net net-benefit differences versus treat-all were −0.007 (95% CI, −0.023 to 0.005) and 0.004 (−0.018 to 0.023), respectively (**Supplementary Table S25**; **Figure 3B**). The difference was 0.044 (0.014–0.071) at 0.30 and 0.151 (0.085–0.212) at 0.50. Thus, the model did not outperform the treat-all mathematical reference in the low-threshold range implied by strong aversion to missed resistance. In the groups labelled 2017–2019 (n = 150; event rate, 63.3%) and 2020–2022 (n = 109; 70.6%), Elastic Net AUROCs were 0.808 and 0.863 and slopes were 0.86 and 0.98; LightGBM AUROCs were 0.829 and 0.888 and slopes were 0.97 and 1.23 (**Supplementary Table S26**). These are descriptive anchor-year-group patterns, not calendar-time trends.

## 4 Discussion

In this single-center retrospective analysis of 811 adult ICU admissions from MIMIC-IV with an ICU-onset sterile-site *Enterococcus*-positive culture, an Elastic Net logistic model built on 19 clinical and culture-level predictors achieved a pseudo-temporal-validation AUROC of 0.835 (95% CI, 0.778-0.889), a calibration slope of 0.91 (0.71-1.18), and a calibration-in-the-large of 0.10 (-0.22 to 0.43). Four tree-based comparators yielded slightly higher AUROC point estimates (0.842-0.855), but no pairwise DeLong comparison reached statistical significance after multiple-comparison correction; these comparisons were not equivalence tests. Calibration was model-dependent: LightGBM’s slope and calibration-in-the-large were close to their ideal values, whereas Random Forest, CatBoost, and XGBoost showed under-dispersed probabilities. To our knowledge, this is the first prediction model that simultaneously targets the pre-susceptibility decision window for ICU-onset sterile-site *Enterococcus*-positive cultures, evaluates pseudo-temporal-validation discrimination and calibration jointly on a common feature set, and benchmarks a penalized linear model against four contemporary tree-based alternatives using identical predictors.

Existing VRE and MDRO prediction models have addressed clinically distinct scenarios, including admission screening (Yoon et al., 2012), colonization-based prediction (Butler-Laporte et al., 2020), hematological cohorts (Webb et al., 2017), colonization-persistence risk (Boeing et al., 2021), and composite MDRO endpoints (Wang et al., 2023; Li et al., 2024), so absolute performance metrics are not directly comparable. Webb et al. reported an AUROC of 0.84 in a hematological cohort using a model that incorporated VRE colonization status (Webb et al., 2017); the different population, predictor availability, and endpoint preclude inference about whether species-level identification can substitute for colonization data. Only two of the reviewed models used pseudo-temporal validation (Boeing et al., 2021; Sun et al., 2026), a methodological gap that matters because AMR epidemiology drifts over time. Because MIMIC-IV dates are shifted at the patient level, the labelled anchor-year groups cannot establish a literal calendar-period effect. They provide a pseudo-temporal ordering for validation but may overlap in real calendar time. The 2017–2019 versus 2020–2022 anchor-group analysis is therefore descriptive only (**Supplementary Table S26**).

Within the common 19-predictor set, no tree-based alternative showed a statistically detectable discrimination advantage over Elastic Net. This finding is consistent with Christodoulou et al.’s systematic review of 71 comparative studies (median sample size, 1,250; eight events per predictor), in which no C-statistic difference was observed between machine learning and logistic regression among low-bias studies, whereas apparent machine-learning superiority was confined to studies with methodological weaknesses (Christodoulou et al., 2019). Our development set (n = 552; 335 events; EPV 17.6) falls within their applicable setting. However, because the common predictors were selected by Elastic Net, the comparison may favor the reference model and does not establish how the tree algorithms would perform with independently optimized feature sets drawn from all 141 candidates. In structured, moderate-dimensional clinical data, penalized regression may capture much of the available signal while retaining interpretability, but multicenter data acquisition, calibration monitoring, workflow integration, and algorithm-specific feature development remain relevant priorities.

Discrimination and calibration diverged sharply. XGBoost, Random Forest, and CatBoost showed AUROCs within 0.020 of the best performer but calibration slopes of 2.41, 1.41, and 1.46, respectively, with confidence intervals excluding 1.0, indicating under-dispersed probabilities. Only Elastic Net (0.91) and LightGBM (1.08) had slope intervals containing 1.0. Probability compression can arise through different mechanisms in ensemble models, including averaging and model-specific regularization; the present data do not identify a common causal mechanism across these algorithms (Niculescu-Mizil and Caruana, 2005). As Van Calster and colleagues have argued, calibration is “the Achilles’ heel of predictive analytics” because clinical utility is more sensitive to miscalibration than to modest differences in discrimination (Van Calster et al., 2019). A model with slope 2.41 may overtreat lower-risk patients whose predictions are pulled upward toward the mean and undertreat higher-risk patients whose predictions are pulled downward, depending on the chosen decision threshold. Development-OOF-fitted Platt scaling brought the Random Forest, XGBoost, and CatBoost slopes close to 1.0 and improved Brier scores (**Supplementary Table S21**). Standard recalibration should therefore be considered a required step for probability-based use of these tree models, rather than a basis for preferring a particular algorithm. Local calibration assessment remains necessary at any prospective site because calibration cannot in general be transported unchanged across settings (Van Calster et al., 2016).

The retained predictors were clinically interpretable, although these post-selection penalized estimates should be interpreted as model associations rather than causal effects. The odds of VRE in *E. faecium* isolates were approximately 22-fold those in *E. faecalis* isolates (implied ratio 22.3, calculated from the two indicator ORs relative to their common reference group of other/unspecified species; *E. faecium* OR 5.81, 95% CI 4.13-8.61; *E. faecalis* OR 0.26, 0.17-0.41). This gradient reflects a well-characterized microbiological structure: hospital-adapted CC17 *E. faecium* lineages frequently carry *vanA*- or *vanB*-bearing mobile elements, whereas resistance in *E. faecalis* remains uncommon in most surveillance datasets (Willems et al., 2005; Top et al., 2008). CatBoost SHAP rankings also identified these two species-level features as the leading contributors, and the full-validation ranking was nearly identical to the former 200-admission ranking (Spearman ρ = 0.998). Removing the two species indicators reduced AUROC from 0.835 to 0.657 (**Supplementary Table S19**). Utility is therefore contingent on rapid species identification; centers without timely species results cannot assume comparable performance.

Rifamycin exposure also warrants careful interpretation. Although rifamycins have no direct therapeutic activity against *Enterococcus*, recent work by Turner and colleagues showed that rifaximin, widely prescribed in chronic liver disease, can select for VRE *E. faecium* carrying RpoB substitutions (S491F, G482D, H486Y) that confer cross-resistance to daptomycin through cell-membrane remodeling; in that cohort, recent rifaximin exposure was independently associated with daptomycin-resistant VRE carriage (OR 6.47; 95% CI, 2.34-20.80) (Turner et al., 2024). Our finding that rifamycin exposure was associated with VRE (OR 1.58) is biologically plausible in this context, but it should not be interpreted as proof of this mechanism in MIMIC-IV because isolate-level genotypes and rifamycin indications were unavailable. Clinically, prior rifamycin exposure may identify patients for whom elevated predicted VRE probability should prompt additional attention to the choice of anti-VRE therapy rather than reliance on vancomycin susceptibility alone.

Other retained predictors have plausible but less specific interpretations. Peritoneal-fluid source (OR 1.61) may reflect the selection pressure of broad-spectrum beta-lactam regimens commonly used for intra-abdominal infection, which often have limited activity against *Enterococcus*. The 2017 Surgical Infection Society revised guidelines recommend that empirical therapy for healthcare-associated intra-abdominal infection in patients at risk for VRE be selected according to the likely species and local strains rather than used routinely (Mazuski et al., 2017); a validated, locally calibrated probability estimate might eventually complement this risk assessment. Skin and soft-tissue procedures, nephrotoxic-agent exposure, endoscopic procedures, and renal replacement therapy may primarily index cumulative healthcare exposure and illness complexity rather than specific resistance mechanisms. The inverse association between aspartate aminotransferase and predicted VRE probability remains exploratory. Its direction was stable across imputation strategies, but removing it did not reduce discrimination (**Supplementary Table S24**); independent evaluation is required before any mechanistic interpretation.

At the prespecified Youden index threshold, sensitivity was 0.884 and specificity 0.690; nonetheless, 25.0% of validation-set patients classified as low risk actually carried a resistant isolate. The Youden index assigns equal weight to sensitivity and specificity and does not encode the asymmetric clinical costs of delayed effective anti-VRE therapy (Ou et al., 2025) versus unnecessary linezolid exposure with its downstream toxicity and selection risks (Cazavet et al., 2020; Misiakou et al., 2023). The supplementary thresholds selected to target specificity or NPV of at least 0.95 in development cross-validation did not consistently retain those properties in pseudo-temporal validation (**Supplementary Table S10**). For Elastic Net, the development-selected rule-out threshold yielded a validation NPV of only 0.818 and therefore cannot justify withholding anti-VRE therapy. These results support evaluating a tiered framework prospectively rather than adopting the reported thresholds: locally validated rule-in and rule-out thresholds could flank an intermediate zone in which rapid molecular testing (*vanA/vanB* PCR, or MALDI-TOF phenotype prediction (Sun et al., 2026)) resolves residual uncertainty. Any such framework should combine model phenotype probability with independent clinical-significance assessment rather than serve as a stand-alone treatment rule.

Extension of these findings is constrained on three fronts. The observed VRE prevalence of 62.5% substantially exceeds contemporary US national estimates (Centers for Disease Control and Prevention, 2019), and between-center prevalence can vary approximately 30-fold in settings with different stewardship and infection-control practices (Cimen et al., 2023), so absolute PPV and NPV should not be assumed to transport. The observed predominance of *E. faecium* among resistant isolates may be consistent with hospital-adapted *E. faecium* ecology, but the absence of isolate genotyping precludes any inference that the local population was CC17-dominant. Centers with different ecology, including many European centers where *vanB* clusters have grown in relative frequency (Hammerum et al., 2024), may exhibit different predictor-outcome relationships. Genotypic information was unavailable in MIMIC-IV: *vanA* and *vanB* could improve resistance-phenotype prediction, while *rpoB* substitutions and other relevant markers could help evaluate daptomycin susceptibility.

Several study-level limitations should also be noted. Cohort eligibility was determined from microbiology records without clinical adjudication of infection versus colonization; the modeled probability is therefore *P*(resistance | positive sterile-site culture), not *P*(resistant infection). MIMIC-IV records specimen collection and final microbiology results but does not reconstruct the exact reporting times of species identification and vancomycin susceptibility. Species was therefore treated as available at the analytical prediction point, but its availability before AST could not be verified for every episode; prospective implementation must confirm predictor availability at the intended decision time. Development-set EPV was 17.6 for VRE but only 11.4 for the smaller VSE class, limiting coefficient precision. Anchor-year date shifting introduces uncertainty into the effective development-to-validation interval, and the observed 5.7-percentage-point prevalence increase cannot be attributed to a specific national or pandemic-era VRE trend. Age-, sex-, and recorded race/ethnicity-stratified analyses were descriptive. The Black, Hispanic, and Asian validation groups each had only 13–17 admissions, precluding fairness or equivalence conclusions and leaving differential performance unresolved. Classification metrics at the Youden index threshold should not be interpreted as treatment thresholds, and the exploratory decision curve analysis does not measure observed changes in prescribing or patient outcomes.

A realistic example of performance attenuation is provided by Ziaian et al. (2025): an XGBoost AMR model had a mean fivefold cross-validation AUROC of 0.896 but an independent held-out test AUROC of 0.786. This single small study is cited as an illustration, not as evidence that all AMR models show the same decline. Together with the prevalence dependence of PPV and NPV (**Supplementary Table S13**), it reinforces that our single-center estimates are not transportability evidence. Independent, adequately sized external validation has not started and remains a prerequisite.

The increase in event prevalence from 60.7% in development to 66.4% in validation, although indexed to de-identified anchor groups rather than literal calendar time, illustrates why deployed models require periodic monitoring. Local programs should track prevalence, calibration intercept and slope, discrimination, and decision-curve performance; recalibration or model updating should be triggered when prespecified tolerances are exceeded.

Cohort selection may also bias transportability. The analysis files did not retain sterile-site *Enterococcus* isolates excluded for missing vancomycin AST, so the requested quantitative included-versus-excluded comparison could not be reconstructed. If AST was more likely to be obtained for clinically severe or resistance-suspected isolates, restriction to tested isolates could enrich resistance and inflate predictive values. In addition, the retained files contain hadm_id and precomputed time to culture but not stay_id or ICU timestamps. We therefore cannot re-audit which ICU stay was linked to culture in admissions with multiple ICU stays and do not claim to have done so.

The transferable contribution of this study is an analytical framework: temporal evaluation of VRE risk models on routinely collected clinical and microbiological data, with joint assessment of discrimination, calibration, and exploratory decision curves, rather than the specific fitted coefficients from a single center. Following the three-phase evidence framework articulated by Reilly and Evans (Reilly and Evans, 2006), multicenter external validation with staged calibration updating (Steyerberg, 2019), followed by silent evaluation in the electronic health record and a pragmatic impact trial, defines the required translational pathway.

## 5 Conclusion

In adult ICU admissions with sterile-site *Enterococcus*-positive cultures obtained more than 48 hours after ICU admission, routinely recorded clinical and culture-level data supported prediction of the index isolate’s vancomycin-resistance phenotype, with pseudo-temporal-validation AUROCs of 0.835–0.855. Species identification supplied most discrimination, and tree-model probability calibration improved substantially after development-OOF-fitted Platt scaling. Decision-curve results did not favor model-guided decisions over treat-all at low thresholds. The model does not determine clinical infection and is not a stand-alone treatment rule. Estimates are specific to one high-prevalence center; independent external validation, local recalibration, confirmation of predictor availability, clinical adjudication, periodic updating, and prospective impact evaluation are prerequisites for implementation.

## Patient and public involvement statement

Patients and the public were not involved in the design, conduct, reporting, interpretation, or dissemination of this retrospective de-identified database study.

## Data Availability

Publicly available datasets were analyzed in this study. This data can be found here: https://physionet.org/content/mimiciv/.
