# Supplementary material for: Prediction of vancomycin-resistant phenotype in ICU-onset sterile-site Enterococcus-positive cultures: development and pseudo-temporal validation of a clinical prediction model using MIMIC-IV
Source: Front Cell Infect Microbiol. 2026 Oct 2;16:1935284. doi: 10.3389/fcimb.2026.1935284 (PMC13672234; doi:10.3389/fcimb.2026.1935284)
Supplement: Supplementary file 1 [file Table1.docx]

Supplementary Material

**Contents**

Table S1. Missingness of candidate predictors in the development and pseudo-temporal validation sets.

Table S2. Sensitivity analysis of missing-data strategies using the fixed 19-predictor Elastic Net architecture.

Table S3. Baseline characteristics: development versus pseudo-temporal validation sets.

Table S4. Bootstrap stability analysis of candidate predictors (100 resamples).

Table S5. Development-set feature support and sparse-cell eligibility audit.

Table S6. Final Elastic Net model: coefficients, odds ratios, and selection frequencies.

Table S7. Pairwise DeLong tests for AUROC between models on the pseudo-temporal validation set.

Table S8. Internal validation: bootstrap optimism correction (200 resamples).

Table S9. Calibration metrics for the five models on the pseudo-temporal validation set.

Table S10. Validation-set performance of development-CV-selected high-specificity and high-NPV thresholds.

Table S11. CatBoost SHAP feature importance (mean absolute SHAP values).

Table S12. Subgroup performance by age and sex on the pseudo-temporal validation set.

Table S13. Prevalence-adjusted PPV and NPV at the Youden index threshold (Elastic Net).

Table S14. Variance inflation factors for the 19 final predictors.

Table S15. Hyperparameter search spaces and selected values for all models.

Table S16. Complete Elastic Net Logistic scoring specification.

Table S17. TRIPOD+AI 2024 reporting checklist.

Table S18. TRIPOD+AI for Abstracts reporting checklist.

Table S19. Sensitivity of model performance to species-level identification.

Table S20. Sensitivity analysis forcing time to culture into the model.

Table S21. Development-OOF-fitted recalibration.

Table S22. Descriptive recorded race/ethnicity subgroup performance.

Table S23. Selection-inclusive bootstrap optimism correction.

Table S24. Aspartate aminotransferase drop and imputation-direction sensitivity.

Table S25. Decision-curve net benefit with paired bootstrap confidence intervals.

Table S26. Performance by anchor-year group.

**Supplementary Figures**

Figure S1. Temporal trend in VRE prevalence by anchor year group.

Figure S2. Bootstrap selection frequency for candidate predictors with non-zero coefficients.

Figure S3. Precision-recall curves for the five models on the pseudo-temporal validation set.

Figure S4. Forest plot of AUROC with 95% bootstrap confidence intervals.

Figure S5. Spearman rank correlation matrix of the 19 final predictors.

Figure S6. Recalibration of under-dispersed tree models.

**Supplementary Tables**

**Table S1. Missingness of candidate predictors in the development and pseudo-temporal validation sets.**

| **Variable** | **Dev set N missing** | **Dev set %** | **Validation set N missing** | **Validation set %** |
| --- | --- | --- | --- | --- |
| total bilirubin | 156 | 28.260 | 53 | 20.460 |
| alanine aminotransferase | 156 | 28.260 | 50 | 19.310 |
| aspartate aminotransferase | 154 | 27.900 | 49 | 18.920 |
| lactate | 147 | 26.630 | 49 | 18.920 |
| temperature | 18 | 3.260 | 4 | 1.540 |
| platelet | 7 | 1.270 | 3 | 1.160 |
| potassium | 5 | 0.910 | 3 | 1.160 |
| hemoglobin | 5 | 0.910 | 3 | 1.160 |
| creatinine | 5 | 0.910 | 3 | 1.160 |
| blood urea nitrogen | 5 | 0.910 | 3 | 1.160 |
| sodium | 5 | 0.910 | 3 | 1.160 |
| bicarbonate | 5 | 0.910 | 3 | 1.160 |
| glucose | 5 | 0.910 | 3 | 1.160 |
| diastolic blood pressure | 4 | 0.720 | 0 | 0.000 |
| systolic blood pressure | 4 | 0.720 | 0 | 0.000 |
| respiratory rate | 3 | 0.540 | 0 | 0.000 |
| oxygen saturation | 3 | 0.540 | 0 | 0.000 |
| heart rate | 3 | 0.540 | 0 | 0.000 |
| mean arterial pressure | 3 | 0.540 | 0 | 0.000 |
| Glasgow coma scale | 2 | 0.360 | 0 | 0.000 |
| All other variables (n = 87) | 0 | 0.000 | 0 | 0.000 |

Only candidate predictors with at least one missing value in either set are listed. Percentages are calculated against the development set (n = 552) and the pseudo-temporal validation set (n = 259), respectively. Variables are sorted by descending missingness in the development set.

*Abbreviations:* N, number.

**Table S2. Sensitivity analysis of missing-data strategies using the fixed 19-predictor Elastic Net architecture.**

| **Strategy** | **Analysis set** | **N** | **N** | **AUROC (95% CI)** | **AUPRC (95% CI)** |
| --- | --- | --- | --- | --- | --- |
|  |  | **development** | **validation** |  |  |
| Single iterative imputation | Full development/validation sets | 552 | 259 | 0.835 | 0.894 |
| Median imputation | Full development/validation sets | 552 | 259 | 0.834 | 0.894 |
| Multiple imputation (Rubin-rules pooled) | Full development/validation sets | 552 | 259 | 0.8393 (0.7761–0.8873) | 0.8971 (0.8410–0.9349) |
| Multiple imputation (mean-probability ensemble) | Full development/validation sets | 552 | 259 | 0.841 | 0.898 |
| Complete-case analysis | Complete-case rows only | 386 | 206 | 0.817 | 0.903 |

All analyses used the same 19 final predictors and the fixed Elastic Net architecture (C = 0.281177, l1_ratio = 0.1). The multiple-imputation analysis used 10 posterior-draw iterative imputations; logit-transformed AUROC and AUPRC estimates were combined using Rubin's rules with within-imputation variance estimated from 200 bootstrap resamples of held-out validation predictions. Confidence intervals are shown for the Rubin-rules pooled estimates; separate bootstrap confidence intervals were not computed for the other strategy-specific point estimates. Complete-case differences may reflect sample size, selection, and case-mix changes.

*Abbreviations:* AUROC, area under the receiver operating characteristic curve; AUPRC, area under the precision-recall curve; CI, confidence interval.

**Table S3. Baseline characteristics of the cohort by development versus pseudo-temporal validation set.**

| **Variable** | **Level** | **Overall (n = 811)** | **Missing** | **Development (n = 552)** | **Validation (n = 259)** | **P value** | **SMD** |
| --- | --- | --- | --- | --- | --- | --- | --- |
| age |  | 61 (52–70) | 0 | 62 (52–70) | 61 (52.5–70.5) | 0.994 | −0.01 |
| time to culture (hours) |  | 315 (155–574) | 0 | 295 (146–552) | 352 (176–599) | 0.105 | 0.050 |
| platelet |  | 156 (81.3–245) | 10 | 158 (84.8–248) | 151 (73.3–230) | 0.280 | −0.09 |
| hemoglobin |  | 9.0 (7.89–10.3) | 8 | 9.1 (8.08–10.4) | 8.57 (7.58–10.1) | <0.001 | −0.16 |
| creatinine |  | 1.25 (0.8–2.37) | 8 | 1.2 (0.8–2.3) | 1.3 (0.8–2.6) | 0.437 | 0.040 |
| blood urea nitrogen |  | 26.5 (16.5–44) | 8 | 26.3 (16.5–42.9) | 27.3 (16.4–46) | 0.907 | 0.000 |
| sodium |  | 138 (134–140) | 8 | 138 (134–140) | 138 (134–142) | 0.309 | 0.070 |
| potassium |  | 4.2 (3.83–4.7) | 8 | 4.2 (3.81–4.66) | 4.26 (3.86–4.75) | 0.180 | 0.100 |
| bicarbonate |  | 21.7 (18.4–24.4) | 8 | 22 (18.5–24.5) | 21 (18–24) | 0.123 | −0.11 |
| glucose |  | 135 (108–175) | 8 | 131 (105–170) | 142 (115–184) | 0.002 | 0.210 |
| total bilirubin |  | 1.3 (0.6–4.29) | 209 | 1.3 (0.7–4.1) | 1.3 (0.5–5.65) | 0.863 | 0.150 |
| alanine aminotransferase |  | 30 (17–80) | 206 | 29.8 (16.9–77.1) | 31.3 (17.5–82) | 0.517 | 0.150 |
| aspartate aminotransferase |  | 53 (28–129) | 203 | 50 (27.6–120) | 58.2 (29.2–162) | 0.223 | 0.120 |
| lactate |  | 2.1 (1.4–3.29) | 196 | 2.0 (1.4–3.22) | 2.2 (1.41–3.63) | 0.374 | 0.070 |
| heart rate |  | 92.1 (80.4–103) | 3 | 91.8 (79.9–103) | 93.9 (82.3–105) | 0.053 | 0.130 |
| systolic blood pressure |  | 110 (103–122) | 4 | 111 (103–122) | 110 (102–120) | 0.715 | −0.02 |
| diastolic blood pressure |  | 60.9 (53.7–66.9) | 4 | 59.9 (53.0–66.8) | 61.7 (54.7–67.4) | 0.051 | 0.150 |
| mean arterial pressure |  | 74.6 (68.7–81.2) | 3 | 73.6 (67.8–80.9) | 75.7 (71.2–81.4) | 0.004 | 0.190 |
| respiratory rate |  | 19.4 (17.0–22.9) | 3 | 19.1 (16.8–22.4) | 20.3 (17.4–23.5) | 0.003 | 0.200 |
| temperature |  | 36.8 (36.6–37.1) | 22 | 36.8 (36.5–37.1) | 36.8 (36.7–37.2) | <0.001 | 0.250 |
| oxygen saturation |  | 97.2 (95.8–98.6) | 3 | 97.3 (95.8–98.7) | 97.1 (95.7–98.3) | 0.048 | −0.06 |
| SOFA score |  | 8 (5–12) | 0 | 8 (5–12) | 9 (5–13) | 0.254 | 0.080 |
| SAPS II score |  | 42 (33.5–53) | 0 | 42 (33–53) | 42 (34–52.5) | 0.933 | 0.000 |
| Glasgow coma scale |  | 15 (15–15) | 2 | 15 (15–15) | 15 (15–15) | 0.750 | −0.02 |
| specimen source | ASPIRATE | 2 (0.2) | 0 | 1 (0.2) | 1 (0.4) | 0.009 | 0.040 |
| specimen source | BIOPSY | 2 (0.2) | 0 | 2 (0.4) | 0 (0.0) |  | −0.09 |
| specimen source | BLOOD CULTURE | 467 (57.6) | 0 | 302 (54.7) | 165 (63.7) |  | 0.180 |
| specimen source | BLOOD CULTURE (MYCO/F LYTIC BOTTLE) | 5 (0.6) | 0 | 3 (0.5) | 2 (0.8) |  | 0.030 |
| specimen source | CSF; SPINAL FLUID | 3 (0.4) | 0 | 1 (0.2) | 2 (0.8) |  | 0.090 |
| specimen source | DIALYSIS FLUID | 2 (0.2) | 0 | 1 (0.2) | 1 (0.4) |  | 0.040 |
| specimen source | FLUID RECEIVED IN BLOOD CULTURE BOTTLES | 20 (2.5) | 0 | 12 (2.2) | 8 (3.1) |  | 0.060 |
| specimen source | FLUID, OTHER | 34 (4.2) | 0 | 31 (5.6) | 3 (1.2) |  | −0.25 |
| specimen source | FOREIGN BODY | 7 (0.9) | 0 | 5 (0.9) | 2 (0.8) |  | −0.01 |
| specimen source | JOINT FLUID | 1 (0.1) | 0 | 0 (0.0) | 1 (0.4) |  | 0.090 |
| specimen source | PERITONEAL FLUID | 110 (13.6) | 0 | 88 (15.9) | 22 (8.5) |  | −0.23 |
| specimen source | PLEURAL FLUID | 16 (2.0) | 0 | 8 (1.4) | 8 (3.1) |  | 0.110 |
| specimen source | TISSUE | 142 (17.5) | 0 | 98 (17.8) | 44 (17.0) |  | −0.02 |
| hypertension | Yes | 236 (29.1) | 0 | 165 (29.9) | 71 (27.4) | 0.507 | −0.05 |
| acute kidney injury | Yes | 496 (61.2) | 0 | 323 (58.5) | 173 (66.8) | 0.025 | 0.170 |
| cirrhosis | Yes | 46 (5.7) | 0 | 26 (4.7) | 20 (7.7) | 0.103 | 0.120 |
| hepatitis | Yes | 28 (3.5) | 0 | 27 (4.9) | 1 (0.4) | <0.001 | −0.28 |
| heart failure | Yes | 241 (29.7) | 0 | 171 (31.0) | 70 (27.0) | 0.284 | −0.09 |
| pneumonia | Yes | 84 (10.4) | 0 | 41 (7.4) | 43 (16.6) | <0.001 | 0.290 |
| coronary artery disease | Yes | 202 (24.9) | 0 | 143 (25.9) | 59 (22.8) | 0.384 | −0.07 |
| myocardial infarction | Yes | 70 (8.6) | 0 | 51 (9.2) | 19 (7.3) | 0.422 | −0.07 |
| stroke | Yes | 47 (5.8) | 0 | 29 (5.3) | 18 (6.9) | 0.337 | 0.070 |
| hyperlipidemia | Yes | 248 (30.6) | 0 | 175 (31.7) | 73 (28.2) | 0.327 | −0.08 |
| chronic kidney disease | Yes | 241 (29.7) | 0 | 179 (32.4) | 62 (23.9) | 0.014 | −0.19 |
| chronic obstructive pulmonary disease | Yes | 8 (1.0) | 0 | 8 (1.4) | 0 (0.0) | 0.061 | −0.17 |
| diabetes | Yes | 115 (14.2) | 0 | 112 (20.3) | 3 (1.2) | <0.001 | −0.65 |
| aminoglycoside | Yes | 16 (2.0) | 0 | 13 (2.4) | 3 (1.2) | 0.416 | −0.09 |
| aztreonam | Yes | 16 (2.0) | 0 | 15 (2.7) | 1 (0.4) | 0.028 | −0.19 |
| carbapenem | Yes | 92 (11.3) | 0 | 70 (12.7) | 22 (8.5) | 0.096 | −0.14 |
| cephalosporin | Yes | 266 (32.8) | 0 | 158 (28.6) | 108 (41.7) | <0.001 | 0.280 |
| clindamycin | Yes | 9 (1.1) | 0 | 6 (1.1) | 3 (1.2) | 1.000 | 0.010 |
| daptomycin | Yes | 59 (7.3) | 0 | 36 (6.5) | 23 (8.9) | 0.247 | 0.090 |
| fidaxomicin | Yes | 1 (0.1) | 0 | 1 (0.2) | 0 (0.0) | 1.000 | −0.06 |
| fluoroquinolone | Yes | 111 (13.7) | 0 | 83 (15.0) | 28 (10.8) | 0.125 | −0.13 |
| isoniazid | Yes | 3 (0.4) | 0 | 1 (0.2) | 2 (0.8) | 0.241 | 0.090 |
| linezolid | Yes | 36 (4.4) | 0 | 26 (4.7) | 10 (3.9) | 0.715 | −0.04 |
| macrolide | Yes | 33 (4.1) | 0 | 21 (3.8) | 12 (4.6) | 0.572 | 0.040 |
| metronidazole | Yes | 180 (22.2) | 0 | 124 (22.5) | 56 (21.6) | 0.856 | −0.02 |
| mupirocin | Yes | 17 (2.1) | 0 | 14 (2.5) | 3 (1.2) | 0.294 | −0.10 |
| nitrofurantoin | Yes | 2 (0.2) | 0 | 2 (0.4) | 0 (0.0) | 1.000 | −0.09 |
| penicillin | Yes | 48 (5.9) | 0 | 33 (6.0) | 15 (5.8) | 1.000 | −0.01 |
| piperacillin tazobactam | Yes | 113 (13.9) | 0 | 83 (15.0) | 30 (11.6) | 0.194 | −0.10 |
| polymyxin bacitracin | Yes | 51 (6.3) | 0 | 25 (4.5) | 26 (10.0) | 0.005 | 0.210 |
| rifamycin | Yes | 97 (12.0) | 0 | 57 (10.3) | 40 (15.4) | 0.048 | 0.150 |
| silver sulfadiazine | Yes | 1 (0.1) | 0 | 1 (0.2) | 0 (0.0) | 1.000 | −0.06 |
| sulfamethoxazole–trimethoprim | Yes | 69 (8.5) | 0 | 52 (9.4) | 17 (6.6) | 0.224 | −0.11 |
| tetracycline | Yes | 16 (2.0) | 0 | 9 (1.6) | 7 (2.7) | 0.294 | 0.070 |
| vancomycin | Yes | 236 (29.1) | 0 | 172 (31.2) | 64 (24.7) | 0.068 | −0.14 |
| abdominal surgery | Yes | 402 (49.6) | 0 | 273 (49.5) | 129 (49.8) | 0.940 | 0.010 |
| organ transplantation | Yes | 65 (8.0) | 0 | 43 (7.8) | 22 (8.5) | 0.782 | 0.030 |
| central venous catheterization | Yes | 417 (51.4) | 0 | 247 (44.7) | 170 (65.6) | <0.001 | 0.430 |
| percutaneous drainage | Yes | 488 (60.2) | 0 | 299 (54.2) | 189 (73.0) | <0.001 | 0.400 |
| mechanical ventilation procedure | Yes | 464 (57.2) | 0 | 312 (56.5) | 152 (58.7) | 0.594 | 0.040 |
| renal replacement therapy procedure | Yes | 259 (31.9) | 0 | 168 (30.4) | 91 (35.1) | 0.196 | 0.100 |
| cardiothoracic surgery | Yes | 229 (28.2) | 0 | 131 (23.7) | 98 (37.8) | <0.001 | 0.310 |
| neurosurgery | Yes | 84 (10.4) | 0 | 58 (10.5) | 26 (10.0) | 0.902 | −0.02 |
| orthopedic procedure | Yes | 604 (74.5) | 0 | 420 (76.1) | 184 (71.0) | 0.142 | −0.11 |
| endoscopic procedure | Yes | 443 (54.6) | 0 | 285 (51.6) | 158 (61.0) | 0.013 | 0.190 |
| hepatobiliary procedure | Yes | 212 (26.1) | 0 | 142 (25.7) | 70 (27.0) | 0.732 | 0.030 |
| skin and soft-tissue procedure | Yes | 214 (26.4) | 0 | 138 (25.0) | 76 (29.3) | 0.200 | 0.100 |
| urologic procedure | Yes | 163 (20.1) | 0 | 134 (24.3) | 29 (11.2) | <0.001 | −0.35 |
| peripheral vascular procedure | Yes | 133 (16.4) | 0 | 76 (13.8) | 57 (22.0) | 0.004 | 0.220 |
| trauma | Yes | 100 (12.3) | 0 | 74 (13.4) | 26 (10.0) | 0.208 | −0.10 |
| head trauma | Yes | 13 (1.6) | 0 | 9 (1.6) | 4 (1.5) | 1.000 | −0.01 |
| spinal trauma | Yes | 15 (1.8) | 0 | 7 (1.3) | 8 (3.1) | 0.093 | 0.120 |
| thoracic trauma | Yes | 23 (2.8) | 0 | 14 (2.5) | 9 (3.5) | 0.498 | 0.050 |
| abdominal trauma | Yes | 15 (1.8) | 0 | 11 (2.0) | 4 (1.5) | 0.785 | −0.03 |
| extremity trauma | Yes | 45 (5.5) | 0 | 29 (5.3) | 16 (6.2) | 0.623 | 0.040 |
| malignancy | Yes | 231 (28.5) | 0 | 165 (29.9) | 66 (25.5) | 0.211 | −0.10 |
| *enterococcus species* | *FAECALIS* | 128 (15.8) | 0 | 84 (15.2) | 44 (17.0) | <0.001 | 0.050 |
| *enterococcus species* | *FAECIUM* | 428 (52.8) | 0 | 267 (48.4) | 161 (62.2) |  | 0.280 |
| *enterococcus species* | OTHER | 10 (1.2) | 0 | 5 (0.9) | 5 (1.9) |  | 0.090 |
| *enterococcus species* | SP | 245 (30.2) | 0 | 196 (35.5) | 49 (18.9) |  | −0.38 |
| male | Yes | 490 (60.4) | 0 | 332 (60.1) | 158 (61.0) | 0.878 | 0.020 |
| white | Yes | 529 (65.2) | 0 | 378 (68.5) | 151 (58.3) | 0.006 | −0.21 |
| black | Yes | 83 (10.2) | 0 | 66 (12.0) | 17 (6.6) | 0.018 | −0.19 |
| hispanic | Yes | 40 (4.9) | 0 | 27 (4.9) | 13 (5.0) | 1.000 | 0.010 |
| asian | Yes | 36 (4.4) | 0 | 21 (3.8) | 15 (5.8) | 0.205 | 0.090 |
| race other | Yes | 123 (15.2) | 0 | 60 (10.9) | 63 (24.3) | <0.001 | 0.360 |
| married | Yes | 361 (44.5) | 0 | 257 (46.6) | 104 (40.2) | 0.096 | −0.13 |
| single | Yes | 245 (30.2) | 0 | 173 (31.3) | 72 (27.8) | 0.326 | −0.08 |
| widowed | Yes | 78 (9.6) | 0 | 61 (11.1) | 17 (6.6) | 0.055 | −0.16 |
| divorced | Yes | 63 (7.8) | 0 | 42 (7.6) | 21 (8.1) | 0.780 | 0.020 |
| marital unknown | Yes | 64 (7.9) | 0 | 19 (3.4) | 45 (17.4) | <0.001 | 0.470 |
| medicare | Yes | 417 (51.4) | 0 | 311 (56.3) | 106 (40.9) | <0.001 | −0.31 |
| medicaid | Yes | 135 (16.6) | 0 | 70 (12.7) | 65 (25.1) | <0.001 | 0.320 |
| private insurance | Yes | 233 (28.7) | 0 | 155 (28.1) | 78 (30.1) | 0.561 | 0.040 |
| insurance other | Yes | 23 (2.8) | 0 | 14 (2.5) | 9 (3.5) | 0.498 | 0.050 |
| insurance unknown | Yes | 3 (0.4) | 0 | 2 (0.4) | 1 (0.4) | 1.000 | 0.000 |
| mechanical ventilation | Yes | 650 (80.1) | 0 | 445 (80.6) | 205 (79.2) | 0.638 | −0.04 |
| renal replacement therapy | Yes | 130 (16.0) | 0 | 83 (15.0) | 47 (18.1) | 0.261 | 0.080 |
| invasive catheter | Yes | 557 (68.7) | 0 | 418 (75.7) | 139 (53.7) | <0.001 | −0.47 |
| intravenous antibiotics | Yes | 664 (81.9) | 0 | 449 (81.3) | 215 (83.0) | 0.625 | 0.040 |
| vasopressors | Yes | 303 (37.4) | 0 | 222 (40.2) | 81 (31.3) | 0.016 | −0.19 |
| corticosteroids | Yes | 217 (26.8) | 0 | 139 (25.2) | 78 (30.1) | 0.148 | 0.110 |
| immunosuppressants | Yes | 94 (11.6) | 0 | 68 (12.3) | 26 (10.0) | 0.410 | −0.07 |
| chemotherapy radiotherapy | Yes | 11 (1.4) | 0 | 5 (0.9) | 6 (2.3) | 0.115 | 0.110 |
| nephrotoxic agents | Yes | 517 (63.7) | 0 | 354 (64.1) | 163 (62.9) | 0.754 | −0.02 |

Continuous variables are presented as median (interquartile range); categorical variables as n (%). P values were obtained from Wilcoxon rank-sum tests for continuous variables, Fisher's exact tests for 2×2 categorical tables, and omnibus Pearson chi-squared tests for multilevel categorical variables. A multilevel variable's omnibus P value is shown once, on its first displayed level. Standardized mean differences (SMDs) are the primary descriptors of difference magnitude.

*Abbreviations:* SMD, standardized mean difference; SOFA, Sequential Organ Failure Assessment; VRE, vancomycin-resistant *Enterococcus*; VSE, vancomycin-susceptible *Enterococcus*.

**Table S4. Bootstrap stability analysis of candidate predictors (100 resamples of the development set).**

| **Feature** | **Selection frequency** | **Selection count (out of 100)** | **Coefficient in candidate-level selector** | **Sign consistency** | **Retained in final model** |
| --- | --- | --- | --- | --- | --- |
| *E. faecium* | 1.000 | 100 | 0.839 | 1.000 | Yes |
| *E. faecalis* | 1.000 | 100 | −0.502 | 1.000 | Yes |
| skin and soft-tissue procedure | 0.980 | 98 | 0.167 | 1.000 | Yes |
| nephrotoxic agents | 0.900 | 90 | 0.159 | 1.000 | Yes |
| endoscopic procedure | 0.890 | 89 | 0.124 | 1.000 | Yes |
| rifamycin | 0.880 | 88 | 0.124 | 1.000 | Yes |
| widowed | 0.830 | 83 | −0.138 | 1.000 | Yes |
| SOFA score | 0.820 | 82 | 0.132 | 1.000 | Yes |
| peritoneal fluid | 0.810 | 81 | 0.120 | 1.000 | Yes |
| aspartate aminotransferase | 0.770 | 77 | −0.139 | 0.990 | Yes |
| renal replacement therapy procedure | 0.750 | 75 | 0.083 | 1.000 | Yes |
| mechanical ventilation | 0.700 | 70 | −0.080 | 1.000 | Yes |
| vancomycin | 0.660 | 66 | 0.055 | 1.000 | Yes |
| sulfamethoxazole–trimethoprim | 0.650 | 65 | −0.047 | 1.000 | Yes |
| tissue | 0.640 | 64 | −0.054 | 1.000 | Yes |
| percutaneous drainage | 0.560 | 56 | 0.016 | 1.000 | Yes |
| corticosteroids | 0.540 | 54 | 0.033 | 1.000 | Yes |
| temperature | 0.510 | 51 | −0.018 | 1.000 | Yes |
| hyperlipidemia | 0.500 | 50 | −0.008 | 1.000 | Yes |
| — | — | — | — | — | — |
| linezolid | 0.780 | 78 | 0.077 | 1.000 | No |
| foreign body | 0.660 | 66 | −0.055 | 1.000 | No |
| mupirocin | 0.650 | 65 | −0.046 | 1.000 | No |
| head trauma | 0.620 | 62 | −0.043 | 1.000 | No |
| biopsy | 0.610 | 61 | 0.072 | 1.000 | No |
| dialysis fluid | 0.590 | 59 | −0.032 | 1.000 | No |
| silver sulfadiazine | 0.580 | 58 | −0.071 | 1.000 | No |
| macrolide | 0.570 | 57 | 0.035 | 1.000 | No |
| aminoglycoside | 0.540 | 54 | 0.049 | 1.000 | No |
| hepatobiliary procedure | 0.490 | 49 | −0.024 | 1.000 | No |
| total bilirubin | 0.480 | 48 | 0.000 | 1.000 | No |
| pleural fluid | 0.470 | 47 | 0.008 | 0.980 | No |
| hemoglobin missing | 0.460 | 46 | 0.000 | 1.000 | No |
| creatinine missing | 0.460 | 46 | 0.000 | 1.000 | No |
| blood urea nitrogen missing | 0.460 | 46 | 0.000 | 1.000 | No |
| sodium missing | 0.460 | 46 | 0.000 | 1.000 | No |
| potassium missing | 0.460 | 46 | 0.000 | 1.000 | No |
| bicarbonate missing | 0.460 | 46 | 0.000 | 1.000 | No |
| glucose missing | 0.460 | 46 | 0.000 | 1.000 | No |
| invasive catheter | 0.450 | 45 | 0.012 | 1.000 | No |
| cardiothoracic surgery | 0.450 | 45 | −0.001 | 1.000 | No |
| metronidazole | 0.450 | 45 | 0.000 | 1.000 | No |
| renal replacement therapy | 0.430 | 43 | 0.016 | 1.000 | No |
| hypertension | 0.420 | 42 | 0.000 | 0.980 | No |
| vasopressors | 0.400 | 40 | 0.000 | 1.000 | No |
| SAPS II score | 0.400 | 40 | 0.000 | 1.000 | No |
| All other variables (n = 96) | <0.40 | — | — | — | No |

Bootstrap selection frequency is the proportion of 100 resamples in which the predictor received a non-zero coefficient. Stable predictors required a selection frequency ≥ 0.50 and a non-zero coefficient in the candidate-level selector. The coefficient shown is from that candidate-level selection model, before support screening. Table S6 reports coefficients from the 19-predictor final model refitted after support screening and hyperparameter retuning; coefficients can therefore differ because the predictor set and regularization strength changed. The Final_Selected column additionally required adequate development-set support; the support audit is reported in Table S5.

**Table S5. Stable binary predictors excluded for inadequate development-set support.**

Binary and one-hot encoded predictors required at least 20 observations with value 1 and at least 5 observations in every predictor (0/1) by outcome (VSE/VRE) cell.

| **Feature** | **Selection frequency** | **N present** | **Present/VSE** | **Present/VRE** | **Minimum 2×2 cell** | **Eligible** |
| --- | --- | --- | --- | --- | --- | --- |
| linezolid | 0.780 | 26 | 4 | 22 | 4 | No |
| foreign body | 0.660 | 5 | 4 | 1 | 1 | No |
| mupirocin | 0.650 | 14 | 8 | 6 | 6 | No |
| head trauma | 0.620 | 9 | 7 | 2 | 2 | No |
| biopsy | 0.610 | 2 | 0 | 2 | 0 | No |
| dialysis fluid | 0.590 | 1 | 1 | 0 | 0 | No |
| silver sulfadiazine | 0.580 | 1 | 1 | 0 | 0 | No |
| macrolide | 0.570 | 21 | 4 | 17 | 4 | No |
| aminoglycoside | 0.540 | 13 | 2 | 11 | 2 | No |

The complete 141-variable eligibility audit is submitted separately as Supplementary Data File S1 (CSV). Screening used only the development set; the pseudo-temporal validation set was not consulted.

**Table S6. Final Elastic Net Logistic model: coefficients, odds ratios, and bootstrap selection frequencies.**

| **Feature** | **Coefficient** | **Odds ratio** | **95% CI** | **Selection frequency** |
| --- | --- | --- | --- | --- |
| *E. faecium* | 1.760 | 5.810 | 4.13–8.61 | 1.000 |
| *E. faecalis* | −1.333 | 0.260 | 0.17–0.41 | 1.000 |
| skin and soft-tissue procedure | 0.596 | 1.810 | 1.21–2.71 | 0.980 |
| nephrotoxic agents | 0.438 | 1.550 | 1.06–2.29 | 0.900 |
| endoscopic procedure | 0.329 | 1.390 | 1.00–1.98 | 0.890 |
| rifamycin | 0.457 | 1.580 | 1.03–2.43 | 0.880 |
| widowed | −0.542 | 0.580 | 0.36–0.95 | 0.830 |
| SOFA score | 0.267 | 1.310 | 1.07–1.61 | 0.820 |
| peritoneal fluid | 0.477 | 1.610 | 1.07–2.46 | 0.810 |
| aspartate aminotransferase | −0.266 | 0.770 | 0.61–0.95 | 0.770 |
| renal replacement therapy procedure | 0.274 | 1.310 | 0.92–2.01 | 0.750 |
| mechanical ventilation | −0.440 | 0.640 | 0.41–1.00 | 0.700 |
| vancomycin | 0.342 | 1.410 | 1.00–2.16 | 0.660 |
| sulfamethoxazole–trimethoprim | −0.440 | 0.640 | 0.38–1.09 | 0.650 |
| tissue | −0.308 | 0.730 | 0.47–1.06 | 0.640 |
| percutaneous drainage | 0.158 | 1.170 | 0.85–1.73 | 0.560 |
| corticosteroids | 0.245 | 1.280 | 0.89–2.05 | 0.540 |
| temperature | −0.135 | 0.870 | 0.71–1.06 | 0.510 |
| hyperlipidemia | −0.184 | 0.830 | 0.57–1.12 | 0.500 |

Coefficients are from the final Elastic Net Logistic model fitted to the full development set (n = 552) using 19 retained predictors. Odds ratios for continuous predictors are per one-standard-deviation increase, and odds ratios for ordinary binary predictors are for presence versus absence. For the retained categorical indicators, the species reference group comprised other Enterococcus species and Enterococcus spp. (species not specified), and the specimen-source reference group comprised all sources other than peritoneal fluid and tissue: aspirate, biopsy, blood culture, myco/F lytic blood culture, cerebrospinal fluid, dialysis fluid, fluid received in blood-culture bottles, other fluid, foreign body, and pleural fluid. The 95% confidence intervals are bootstrap percentile intervals from 1,000 resamples. Estimates are descriptive quantities conditional on support screening, feature selection, and penalized fitting.

*Abbreviations:* CI, confidence interval; SD, standard deviation; VRE, vancomycin-resistant *Enterococcus*; VSE, vancomycin-susceptible *Enterococcus*.

**Table S7. Pairwise DeLong tests of AUROC differences between models on the pseudo-temporal validation set.**

| **Model A** | **Model B** | **AUROC A** | **AUROC B** | **ΔAUROC** | **DeLong Z** | **P value** | **Bonferroni- adjusted P** | **BH- adjusted P** |
| --- | --- | --- | --- | --- | --- | --- | --- | --- |
| Elastic Net Logistic | Random Forest | 0.835 | 0.848 | −0.013 | −1.08 | 0.279 | 1.000 | 0.398 |
| Elastic Net Logistic | XGBoost | 0.835 | 0.842 | −0.008 | −0.58 | 0.561 | 1.000 | 0.623 |
| Elastic Net Logistic | LightGBM | 0.835 | 0.853 | −0.019 | −1.58 | 0.114 | 1.000 | 0.227 |
| Elastic Net Logistic | CatBoost | 0.835 | 0.855 | −0.021 | −1.71 | 0.087 | 0.867 | 0.227 |
| Random Forest | XGBoost | 0.848 | 0.842 | 0.005 | 1.180 | 0.238 | 1.000 | 0.397 |
| Random Forest | LightGBM | 0.848 | 0.853 | −0.006 | −0.87 | 0.386 | 1.000 | 0.483 |
| Random Forest | CatBoost | 0.848 | 0.855 | −0.008 | −1.61 | 0.107 | 1.000 | 0.227 |
| XGBoost | LightGBM | 0.842 | 0.853 | −0.011 | −1.63 | 0.104 | 1.000 | 0.227 |
| XGBoost | CatBoost | 0.842 | 0.855 | −0.013 | −1.99 | 0.047 | 0.466 | 0.227 |
| LightGBM | CatBoost | 0.853 | 0.855 | −0.002 | −0.29 | 0.772 | 1.000 | 0.772 |

DeLong tests compared AUROC values on the same pseudo-temporal validation set predictions (n = 259). ΔAUROC = AUROC of model B − AUROC of model A. Multiple testing across the 10 pairwise comparisons was adjusted using the Bonferroni method and the Benjamini–Hochberg false-discovery-rate procedure. No pair reached statistical significance at α = 0.05 after either correction.

*Abbreviations:* AUROC, area under the receiver operating characteristic curve; BH, Benjamini–Hochberg; CI, confidence interval.

**Table S8. Internal validation by bootstrap optimism correction (200 resamples of the development set).**

| **Model** | **Apparent AUROC** | **Mean optimism** | **Optimism SD** | **Optimism-corrected AUROC** | **Pseudo-temporal validation AUROC** | **Bootstrap resamples** |
| --- | --- | --- | --- | --- | --- | --- |
| Elastic Net Logistic | 0.870 | 0.019 | 0.015 | 0.851 | 0.835 | 200 |
| Random Forest | 0.908 | 0.045 | 0.009 | 0.863 | 0.848 | 200 |
| XGBoost | 0.923 | 0.055 | 0.008 | 0.869 | 0.842 | 200 |
| LightGBM | 0.907 | 0.054 | 0.009 | 0.854 | 0.853 | 200 |
| CatBoost | 0.899 | 0.044 | 0.010 | 0.855 | 0.855 | 200 |

Apparent AUROC is the AUROC of the final model evaluated on the full development set on which it was fitted. Mean optimism is the average over 200 bootstrap iterations of (AUROC on bootstrap sample) − (AUROC of bootstrap-fitted model applied to the full development set). Optimism-corrected AUROC = apparent AUROC − mean optimism. The pseudo-temporal validation AUROC is reproduced from the main analysis to allow direct comparison.

*Abbreviations:* AUROC, area under the receiver operating characteristic curve; SD, standard deviation.

**Table S9. Calibration metrics for the five models on the pseudo-temporal validation set.**

| **Model** | **Calibration slope** | **Slope 95% CI** | **Calibration intercept** | **CITL** | **CITL 95% CI** | **ECE** |
| --- | --- | --- | --- | --- | --- | --- |
| Elastic Net Logistic | 0.910 | 0.71–1.18 | 0.140 | 0.100 | −0.22–0.43 | 0.076 |
| Random Forest | 1.410 | 1.08–1.87 | −0.10 | 0.110 | −0.15–0.38 | 0.079 |
| XGBoost | 2.410 | 1.88–3.16 | −0.51 | 0.140 | −0.07–0.41 | 0.153 |
| LightGBM | 1.080 | 0.83–1.41 | 0.000 | 0.040 | −0.26–0.35 | 0.054 |
| CatBoost | 1.460 | 1.14–1.88 | −0.09 | 0.140 | −0.12–0.41 | 0.082 |

The calibration slope and intercept were estimated by regressing the binary outcome on the logit of predicted probabilities; a slope of 1.0 and an intercept of 0.0 indicate perfect calibration. Calibration-in-the-large (CITL) was estimated as the intercept from a binomial generalized linear model with the logit of predicted risk as an offset; a CITL of 0.0 indicates agreement in average predicted and observed risk, and positive values indicate average underprediction. The expected calibration error (ECE) was computed with 10 equal-width bins of predicted probability. The 95% confidence intervals for calibration slope and CITL are bootstrap percentile intervals from 1,000 resamples of the pseudo-temporal validation set.

*Abbreviations:* CI, confidence interval; CITL, calibration-in-the-large; ECE, expected calibration error.

**Table S10. Validation-set performance of thresholds selected to target specificity ≥ 0.95 or NPV ≥ 0.95 in development-set cross-validation.**

| **Model** | **Scenario** | **Threshold** | **Sensitivity** | **Specificity** | **PPV** | **NPV** | **F1** | **TP** | **FP** | **TN** | **FN** |
| --- | --- | --- | --- | --- | --- | --- | --- | --- | --- | --- | --- |
| Elastic Net Logistic | Rule-in (Spec ≥0.95) | 0.882 | 0.384 | 0.920 | 0.904 | 0.430 | 0.539 | 66 | 7 | 80 | 106 |
| Random Forest | Rule-in (Spec ≥0.95) | 0.808 | 0.401 | 0.897 | 0.885 | 0.431 | 0.552 | 69 | 9 | 78 | 103 |
| XGBoost | Rule-in (Spec ≥0.95) | 0.918 | 0.256 | 0.954 | 0.917 | 0.393 | 0.400 | 44 | 4 | 83 | 128 |
| LightGBM | Rule-in (Spec ≥0.95) | 0.917 | 0.297 | 0.943 | 0.911 | 0.404 | 0.447 | 51 | 5 | 82 | 121 |
| CatBoost | Rule-in (Spec ≥0.95) | 0.867 | 0.401 | 0.931 | 0.920 | 0.440 | 0.559 | 69 | 6 | 81 | 103 |
| Elastic Net Logistic | Rule-out (NPV ≥0.95) | 0.191 | 0.965 | 0.310 | 0.735 | 0.818 | 0.834 | 166 | 60 | 27 | 6 |
| Random Forest | Rule-out (NPV ≥0.95) | 0.255 | 0.977 | 0.241 | 0.718 | 0.840 | 0.828 | 168 | 66 | 21 | 4 |
| XGBoost | Rule-out (NPV ≥0.95) | 0.053 | 1.000 | 0.034 | 0.672 | 1.000 | 0.804 | 172 | 84 | 3 | 0 |
| LightGBM | Rule-out (NPV ≥0.95) | 0.063 | 0.988 | 0.046 | 0.672 | 0.667 | 0.800 | 170 | 83 | 4 | 2 |
| CatBoost | Rule-out (NPV ≥0.95) | 0.087 | 1.000 | 0.023 | 0.669 | 1.000 | 0.802 | 172 | 85 | 2 | 0 |

Thresholds were selected on development-set cross-validated out-of-fold predictions and then applied to the pseudo-temporal validation set predictions (n = 259, of whom 172 were VRE). Rule-in and rule-out labels describe supplementary statistical operating points based on specificity or NPV and are not recommendations to initiate or withhold treatment. Classification metrics at the prespecified Youden index threshold are reported for the Elastic Net Logistic model in the main Results.

*Abbreviations:* FN, false negatives; FP, false positives; NPV, negative predictive value; PPV, positive predictive value; TN, true negatives; TP, true positives; VRE, vancomycin-resistant *Enterococcus*.

**Table S11. CatBoost feature importance ranked by mean absolute SHAP value in all 259 pseudo-temporal-validation admissions.**

| **Rank** | **Feature** | **Mean \|SHAP value\|** |
| --- | --- | --- |
| 1 | *E. faecium* | 0.715 |
| 2 | *E. faecalis* | 0.324 |
| 3 | SOFA score | 0.114 |
| 4 | nephrotoxic agents | 0.094 |
| 5 | endoscopic procedure | 0.066 |
| 6 | aspartate aminotransferase | 0.064 |
| 7 | vancomycin | 0.060 |
| 8 | percutaneous drainage | 0.049 |
| 9 | renal replacement therapy procedure | 0.046 |
| 10 | skin and soft-tissue procedure | 0.044 |
| 11 | temperature | 0.044 |
| 12 | rifamycin | 0.034 |
| 13 | corticosteroids | 0.032 |
| 14 | widowed | 0.025 |
| 15 | mechanical ventilation | 0.023 |
| 16 | peritoneal fluid | 0.014 |
| 17 | hyperlipidemia | 0.013 |
| 18 | tissue | 0.011 |
| 19 | sulfamethoxazole–trimethoprim | 0.005 |

SHAP (SHapley Additive exPlanations) values were computed for the CatBoost model using TreeSHAP in all 259 pseudo-temporal-validation admissions. The full validation set was used for both the numerical ranking and the beeswarm plot in Figure 4B. The mean absolute SHAP value summarizes each feature's average contribution magnitude to the model's log-odds output. The full-sample ranking was nearly identical to the original 200-admission ranking (Spearman ρ = 0.998; *P* < 0.001).

*Abbreviation:* SHAP, SHapley Additive exPlanations.

**Table S12. Discrimination by demographic subgroup on the pseudo-temporal validation set.**

| **Model** | **Subgroup** | **N** | **VRE events** | **Event rate** | **AUROC** | **AUPRC** |
| --- | --- | --- | --- | --- | --- | --- |
| Elastic Net Logistic | Age <65 years | 148 | 105 | 0.709 | 0.845 | 0.918 |
| Elastic Net Logistic | Age ≥65 years | 111 | 67 | 0.604 | 0.808 | 0.852 |
| Elastic Net Logistic | Female | 101 | 64 | 0.634 | 0.873 | 0.909 |
| Elastic Net Logistic | Male | 158 | 108 | 0.684 | 0.811 | 0.889 |
| Random Forest | Age <65 years | 148 | 105 | 0.709 | 0.838 | 0.894 |
| Random Forest | Age ≥65 years | 111 | 67 | 0.604 | 0.848 | 0.888 |
| Random Forest | Female | 101 | 64 | 0.634 | 0.878 | 0.890 |
| Random Forest | Male | 158 | 108 | 0.684 | 0.829 | 0.890 |
| XGBoost | Age <65 years | 148 | 105 | 0.709 | 0.839 | 0.889 |
| XGBoost | Age ≥65 years | 111 | 67 | 0.604 | 0.834 | 0.857 |
| XGBoost | Female | 101 | 64 | 0.634 | 0.875 | 0.889 |
| XGBoost | Male | 158 | 108 | 0.684 | 0.821 | 0.876 |
| LightGBM | Age <65 years | 148 | 105 | 0.709 | 0.847 | 0.913 |
| LightGBM | Age ≥65 years | 111 | 67 | 0.604 | 0.845 | 0.887 |
| LightGBM | Female | 101 | 64 | 0.634 | 0.881 | 0.914 |
| LightGBM | Male | 158 | 108 | 0.684 | 0.836 | 0.902 |
| CatBoost | Age <65 years | 148 | 105 | 0.709 | 0.843 | 0.897 |
| CatBoost | Age ≥65 years | 111 | 67 | 0.604 | 0.857 | 0.893 |
| CatBoost | Female | 101 | 64 | 0.634 | 0.885 | 0.901 |
| CatBoost | Male | 158 | 108 | 0.684 | 0.836 | 0.895 |

Subgroup analyses are descriptive; no formal interaction tests were performed. Event rate is the proportion of VRE cases within each subgroup. Subgroup sample sizes (range, 101–158) limit the precision of these estimates.

*Abbreviations:* AUROC, area under the receiver operating characteristic curve; AUPRC, area under the precision-recall curve; VRE, vancomycin-resistant *Enterococcus*.

**Table S13. Prevalence-adjusted PPV and NPV for the Elastic Net Logistic model at the Youden index threshold.**

| **Assumed VRE prevalence** | **PPV** | **NPV** |
| --- | --- | --- |
| 0.050 | 0.130 | 0.991 |
| 0.100 | 0.240 | 0.982 |
| 0.200 | 0.416 | 0.960 |
| 0.300 | 0.550 | 0.933 |
| 0.400 | 0.655 | 0.899 |
| 0.500 | 0.740 | 0.856 |
| 0.625 | 0.826 | 0.781 |
| 0.700 | 0.869 | 0.718 |

PPV and NPV were recalculated using Bayes' theorem while holding the Elastic Net Logistic model's sensitivity (0.884) and specificity (0.690) at the Youden index threshold fixed across assumed prevalence values. These projections assume unchanged operating characteristics and do not establish rule-in, rule-out, or treatment performance in populations with different prevalence or case mix.

*Abbreviations:* NPV, negative predictive value; PPV, positive predictive value; VRE, vancomycin-resistant *Enterococcus*.

**Table S14. Variance inflation factors (VIFs) for the 19 final predictors in the development set.**

| **Feature** | **VIF** |
| --- | --- |
| *E. faecium* | 2.220 |
| *E. faecalis* | 1.820 |
| tissue | 1.710 |
| peritoneal fluid | 1.710 |
| SOFA score | 1.490 |
| vancomycin | 1.430 |
| nephrotoxic agents | 1.420 |
| renal replacement therapy procedure | 1.290 |
| percutaneous drainage | 1.210 |
| skin and soft-tissue procedure | 1.160 |
| rifamycin | 1.140 |
| corticosteroids | 1.130 |
| mechanical ventilation | 1.130 |
| endoscopic procedure | 1.120 |
| aspartate aminotransferase | 1.080 |
| sulfamethoxazole–trimethoprim | 1.070 |
| widowed | 1.060 |
| temperature | 1.060 |
| hyperlipidemia | 1.040 |

Variance inflation factors were computed in the full development set (n = 552) using ordinary least squares regression of each predictor on the remaining 18 predictors. All VIFs were below 2.3, indicating that multicollinearity among the final predictors was within acceptable limits for stable coefficient estimation.

*Abbreviation:* VIF, variance inflation factor.

**Table S15. Hyperparameter search spaces and selected values for all five models.**

Hyperparameters were selected by development-set time-block cross-validation using AUROC as the optimization metric. Elastic Net Logistic and Random Forest used exhaustive grid search; XGBoost and LightGBM used 20 randomized draws, and CatBoost used 15 randomized draws. All randomized searches used seed 42.

| **Model** | **Search method** | **Parameter** | **Candidate values** | **Selected value** |
| --- | --- | --- | --- | --- |
| Elastic Net Logistic | Grid search | C | 50 log-spaced values from 0.001 to 100 | 0.281 |
| Elastic Net Logistic | Grid search | l1_ratio | 0.1, 0.3, 0.5, 0.7, 0.9, 0.95 | 0.100 |
| Random Forest | Grid search | n_estimators | 300, 500 | 500 |
| Random Forest | Grid search | max_depth | 5, 10, None | 5 |
| Random Forest | Grid search | min_samples_leaf | 1, 2, 5 | 5 |
| Random Forest | Grid search | max_features | sqrt, 0.5 | sqrt |
| XGBoost | Randomized search (20 draws) | n_estimators | 100, 200, 400 | 100 |
| XGBoost | Randomized search (20 draws) | max_depth | 3, 5, 7 | 5 |
| XGBoost | Randomized search (20 draws) | learning_rate | 0.01, 0.05, 0.1 | 0.010 |
| XGBoost | Randomized search (20 draws) | subsample | 0.7, 0.9, 1.0 | 0.700 |
| XGBoost | Randomized search (20 draws) | colsample_bytree | 0.7, 0.9, 1.0 | 0.700 |
| XGBoost | Randomized search (20 draws) | reg_lambda | 0.5, 1.0, 2.0 | 2 |
| LightGBM | Randomized search (20 draws) | n_estimators | 100, 200, 400 | 400 |
| LightGBM | Randomized search (20 draws) | num_leaves | 15, 31, 63 | 15 |
| LightGBM | Randomized search (20 draws) | max_depth | 3, 5, −1 | 3 |
| LightGBM | Randomized search (20 draws) | learning_rate | 0.01, 0.05, 0.1 | 0.010 |
| LightGBM | Randomized search (20 draws) | subsample | 0.7, 0.9, 1.0 | 0.700 |
| LightGBM | Randomized search (20 draws) | colsample_bytree | 0.7, 0.9, 1.0 | 1 |
| LightGBM | Randomized search (20 draws) | reg_lambda | 0, 1.0, 2.0 | 1 |
| CatBoost | Randomized search (15 draws) | iterations | 100, 200, 400 | 200 |
| CatBoost | Randomized search (15 draws) | depth | 4, 6, 8 | 4 |
| CatBoost | Randomized search (15 draws) | learning_rate | 0.01, 0.05, 0.1 | 0.010 |
| CatBoost | Randomized search (15 draws) | l2_leaf_reg | 1, 3, 5, 7 | 5 |

Fixed non-default settings were as follows. Elastic Net Logistic: penalty = elasticnet; solver = saga; max_iter = 20,000; random_state = 42. Random Forest: random_state = 42; n_jobs = −1. XGBoost: objective = binary:logistic; eval_metric = auc; tree_method = hist; random_state = 42; verbosity = 0; n_jobs = 1. LightGBM: objective = binary; random_state = 42; verbose = −1; n_jobs = 1. CatBoost: eval_metric = AUC; random_seed = 42; verbose = 0; allow_writing_files = False. Parameters not listed in the table or this note retained the defaults of the software versions reported in the Methods.

*Abbreviations:* AUROC, area under the receiver operating characteristic curve; AUC, area under the curve.

**Table S16. Complete Elastic Net Logistic scoring specification.**

The linear predictor is η = intercept + Σ(β_j × x_j), and the predicted probability is 1 / (1 + exp(−η)). Continuous variables are transformed as x_j = (imputed value − development mean) / development SD. The SD is the population SD used by scikit-learn StandardScaler (ddof = 0). Binary indicators remain on their original 0/1 scale. The coefficients below are from the final model fitted to all 552 development admissions.

| **Term** | **Input coding** | **Coefficient** | **Development mean** | **Development SD** | **Comparator/unit** |
| --- | --- | --- | --- | --- | --- |
| Intercept | Constant | −0.552304 |  |  |  |
| *E. faecium* | *1 = E. faecium; 0 = all other species categories* | 1.760 |  |  | Composite other-species group |
| *E. faecalis* | *1 = E. faecalis; 0 = all other species categories* | −1.333288 |  |  | Composite other-species group |
| skin and soft-tissue procedure | Binary; 1 = present/exposed, 0 = absent/not exposed | 0.596 |  |  | Absence (0) |
| nephrotoxic agents | Binary; 1 = present/exposed, 0 = absent/not exposed | 0.438 |  |  | Absence (0) |
| endoscopic procedure | Binary; 1 = present/exposed, 0 = absent/not exposed | 0.329 |  |  | Absence (0) |
| rifamycin | Binary; 1 = present/exposed, 0 = absent/not exposed | 0.457 |  |  | Absence (0) |
| widowed | Binary; 1 = present/exposed, 0 = absent/not exposed | −0.541934 |  |  | Absence (0) |
| SOFA score | Continuous; standardized after development-set imputation | 0.267 | 8.790 | 4.990 | Per 1-SD increase |
| peritoneal fluid | 1 = peritoneal fluid; 0 = all other specimen-source categories | 0.477 |  |  | Composite other-source group |
| aspartate aminotransferase | Continuous; standardized after development-set imputation | −0.265670 | 240.527 | 681.425 | Per 1-SD increase |
| renal replacement therapy procedure | Binary; 1 = present/exposed, 0 = absent/not exposed | 0.274 |  |  | Absence (0) |
| mechanical ventilation | Binary; 1 = present/exposed, 0 = absent/not exposed | −0.439720 |  |  | Absence (0) |
| vancomycin | Binary; 1 = present/exposed, 0 = absent/not exposed | 0.342 |  |  | Absence (0) |
| sulfamethoxazole–trimethoprim | Binary; 1 = present/exposed, 0 = absent/not exposed | −0.439887 |  |  | Absence (0) |
| tissue | 1 = tissue; 0 = all other specimen-source categories | −0.308023 |  |  | Composite other-source group |
| percutaneous drainage | Binary; 1 = present/exposed, 0 = absent/not exposed | 0.158 |  |  | Absence (0) |
| corticosteroids | Binary; 1 = present/exposed, 0 = absent/not exposed | 0.245 |  |  | Absence (0) |
| temperature | Continuous; standardized after development-set imputation | −0.135291 | 36.797 | 0.558 | Per 1-SD increase |
| hyperlipidemia | Binary; 1 = present/exposed, 0 = absent/not exposed | −0.183561 |  |  | Absence (0) |

No category was explicitly dropped during one-hot encoding. For the retained species indicators, the zero-indicator comparator comprises all other species categories; for the retained specimen-source indicators, it comprises all other specimen-source categories. Continuous values were imputed by the fitted development-set IterativeImputer before standardization; binary values were mode-imputed. Exact application therefore requires the archived preprocessing object in addition to the coefficients shown here.

*Abbreviations:* AST, aspartate aminotransferase; SD, standard deviation; SOFA, Sequential Organ Failure Assessment.

**Table S17. TRIPOD+AI 2024 reporting checklist.**

D = development; E = evaluation. Reporting locations refer to the current manuscript component files and should be updated to final page or line numbers after journal formatting.

| **Section** | **Topic** | **Item** | **Type** | **TRIPOD+AI reporting recommendation** | **Location in this study** |
| --- | --- | --- | --- | --- | --- |
| TITLE | Title | 1 | D; E | Identify the study as developing or evaluating the performance of a multivariable prediction model, the target population, and the outcome to be predicted | Title: identifies model development and temporal evaluation, the ICU sterile-site Enterococcus-positive target population, and vancomycin resistance as the outcome. |
| ABSTRACT | Abstract | 2 | D; E | See TRIPOD+AI for Abstracts checklist | Pending final assembled manuscript: the component files reviewed did not include an abstract; complete the separate TRIPOD+AI for Abstracts checklist before submission. |
| INTRODUCTION | Background | 3a | D; E | Explain the healthcare context (including whether diagnostic or prognostic) and rationale for developing or evaluating the prediction model, including references to existing models | Introduction: describes the pre-susceptibility decision window, VRE burden, diagnostic delay, and limitations of existing VRE/MDRO models. |
|  | Background | 3b | D; E | Describe the target population and the intended purpose of the prediction model in the context of the care pathway, including its intended users (e.g., healthcare professionals, patients, public) | Introduction final paragraphs and Methods—Unit of Analysis: define the target population, prediction point, intended clinician users, and risk-stratification purpose. |
|  | Background | 3c | D; E | Describe any known health inequalities between sociodemographic groups | No VRE-specific healthcare inequality was assumed; limited demographic fairness assessment and single-center representativeness are addressed in Methods and Discussion. |
|  | Objectives | 4 | D; E | Specify the study objectives, including whether the study describes the development or validation of a prediction model (or both) | Introduction final paragraph: objective is development and temporal evaluation of VRE prediction models. |
| METHODS | Data | 5a | D; E | Describe the sources of data separately for the development and evaluation datasets (e.g., randomised trial, cohort, routine care or registry data), the rationale for using these data, and representativeness of the data | Methods—Study Design and Data Source/Pseudo-Temporal Split by Anchor-Year Group: MIMIC-IV v3.0 routine-care data; earlier anchor-year groups for development and later groups for evaluation. |
|  | Data | 5b | D; E | Specify the dates of the collected participant data, including start and end of participant accrual; and, if applicable, end of follow-up | Methods—Study Design and Data Source/Pseudo-Temporal Split by Anchor-Year Group: MIMIC-IV 2008–2022 anchor-year groups, with the limitations of de-identified time stated. |
|  | Participants | 6a | D; E | Specify key elements of the study setting (e.g., primary care, secondary care, general population) including the number and location of centres | Methods—Study Design and Study Population: single US academic medical-center ICU cohort. |
|  | Participants | 6b | D; E | Describe the eligibility criteria for study participants | Methods—Inclusion Criteria and Exclusion Criteria: complete eligibility and index-culture rules. |
|  | Participants | 6c | D; E | Give details of any treatments received, and how they were handled during model development or evaluation, if relevant | Methods—Predictors: antimicrobial exposures, procedures, and organ support before specimen collection were candidate predictors; post-culture information was excluded. |
|  | Data preparation | 7 | D; E | Describe any data pre-processing and quality checking, including whether this was similar across relevant sociodemographic groups | Methods—Missing Data and Preprocessing and Feature Selection: imputation, encoding, missing indicators, scaling, quality/support screening, and leakage controls. |
|  | Outcome | 8a | D; E | Clearly define the outcome that is being predicted and the time horizon, including how and when assessed, the rationale for choosing this outcome, and whether the method of outcome assessment is consistent across sociodemographic groups | Methods—Outcome Definition: laboratory-reported susceptible versus resistant vancomycin phenotype of the index isolate; no prognostic horizon. |
|  | Outcome | 8b | D; E | If outcome assessment requires subjective interpretation, describe the qualifications and demographic characteristics of the outcome assessors | Not applicable: outcome classification used recorded clinical microbiology susceptibility results rather than subjective assessor judgment. |
|  | Outcome | 8c | D; E | Report any actions to blind assessment of the outcome to be predicted | Not applicable: retrospective objective laboratory outcome; the outcome was excluded from all predictors. |
|  | Predictors | 9a | D | Describe the choice of initial predictors (e.g., literature, previous models, all available predictors) and any pre-selection of predictors before model building | Methods—Predictors and Feature Selection: candidate domains, development-only Elastic Net selection, bootstrap stability, sparse-cell screening, and 19-predictor cap. |
|  | Predictors | 9b | D; E | Clearly define all predictors, including how and when they were measured (and any actions to blind assessment of predictors for the outcome and other predictors) | Methods—Predictors and Supplementary Tables S3–S6: predictor definitions, measurement windows, coding, and reference groups. |
|  | Predictors | 9c | D; E | If predictor measurement requires subjective interpretation, describe the qualifications and demographic characteristics of the predictor assessors | Not applicable: predictors were structured EHR and microbiology variables without subjective assessor interpretation. |
|  | Sample size | 10 | D; E | Explain how the study size was arrived at (separately for development and evaluation), and justify that the study size was sufficient to answer the research question. Include details of any sample size calculation | Methods—Feature Selection and Results—Model Development: all 811 eligible admissions were used; development n = 552 (335 events), evaluation n = 259 (172 events), EPV = 17.63 and smaller-class ratio = 11.42. |
|  | Missing data | 11 | D; E | Describe how missing data were handled. Provide reasons for omitting any data | Methods—Missing Data and Sensitivity Analyses; Tables S1–S2: single iterative primary imputation, indicators, median/complete-case checks, and 10-dataset multiple imputation. |
|  | Analytical methods | 12a | D | Describe how the data were used (e.g., for development and evaluation of model performance) in the analysis, including whether the data were partitioned, considering any sample size requirements | Methods—Pseudo-Temporal Split by Anchor-Year Group, Feature Selection, Model Development, and Model Evaluation: nonoverlapping development and held-out temporal-evaluation groups. |
|  | Analytical methods | 12b | D | Depending on the type of model, describe how predictors were handled in the analyses (functional form, rescaling, transformation, or any standardisation) | Methods—Missing Data and Preprocessing/Model Development: coding, one-hot encoding, standardization, and interpretation of continuous and binary predictors. |
|  | Analytical methods | 12c | D | Specify the type of model, rationale, all model-building steps, including any hyperparameter tuning, and method for internal validation | Methods—Feature Selection and Model Development; Tables S4–S6, S8, and S15–S16: five models, tuning, internal validation, final parameters, and scoring specification. |
|  | Analytical methods | 12d | D; E | Describe if and how any heterogeneity in estimates of model parameter values and model performance was handled and quantified across clusters (e.g., hospitals, countries). See TRIPOD-Cluster for additional considerations | Not applicable to multicenter clustering: single-center data. Bootstrap uncertainty and subgroup performance are reported. |
|  | Analytical methods | 12e | D; E | Specify all measures and plots used (and their rationale) to evaluate model performance (e.g., discrimination, calibration, clinical utility) and, if relevant, to compare multiple models | Methods—Model Evaluation through Decision Curve Analysis: AUROC, AUPRC, Brier score, threshold metrics, calibration, DeLong comparisons, DCA, and subgroup analyses. |
|  | Analytical methods | 12f | E | Describe any model updating (e.g., recalibration) arising from the model evaluation, either overall or for particular sociodemographic groups or settings | Methods—Calibration Assessment: post hoc exploratory recalibration trigger and its limitations; no recalibration was performed. |
|  | Analytical methods | 12g | E | For model evaluation, describe how the model predictions were calculated (e.g., formula, code, object, application programming interface) | Methods—Model Development/Evaluation and Table S16: fitted pipelines generated probabilities; full Elastic Net specification and planned code availability are reported. |
|  | Class imbalance | 13 | D; E | If class imbalance methods were used, state why and how this was done, and any subsequent methods to recalibrate the model or the model predictions | No resampling or class-weighting method was used specifically for class imbalance; observed prevalence and threshold-dependent metrics are reported. |
|  | Fairness | 14 | D; E | Describe any approaches that were used to address model fairness and their rationale | Methods—Sensitivity Analyses: descriptive age and sex subgroup performance; no formal fairness intervention or interaction testing. |
|  | Model output | 15 | D | Specify the output of the prediction model (e.g., probabilities, classification). Provide details and rationale for any classification and how the thresholds were identified | Methods—Threshold Selection: probabilistic output; Youden index threshold selected from development out-of-fold predictions, with high-specificity/high-NPV descriptive alternatives. |
|  | Training vs evaluation | 16 | D; E | Identify any differences between the development and evaluation data in healthcare setting, eligibility criteria, outcome, and predictors | Methods—Pseudo-Temporal Split by Anchor-Year Group and Results—Cohort Characteristics; Table S3: same source and eligibility criteria, with differences in outcome and predictor distributions reported. |
|  | Ethical approval | 17 | D; E | Name the institutional research board or ethics committee that approved the study and describe the participant-informed consent or the ethics committee waiver of informed consent | Methods—Study Design and Data Source: BIDMC/MIT approvals and waiver for de-identified data. |
| OPEN SCIENCE | Funding | 18a | D; E | Give the source of funding and the role of the funders for the present study | Not reported in the component files reviewed: add all funding sources and the funders' role before submission. |
|  | Conflicts of interest | 18b | D; E | Declare any conflicts of interest and financial disclosures for all authors | Not reported in the component files reviewed: add competing-interest and financial-disclosure statements for all authors before submission. |
|  | Protocol | 18c | D; E | Indicate where the study protocol can be accessed or state that a protocol was not prepared | No preregistered protocol was prepared; this is disclosed for the post hoc recalibration trigger. |
|  | Registration | 18d | D; E | Provide registration information for the study, including register name and registration number, or state that the study was not registered | Retrospective database study; not prospectively registered. |
|  | Data sharing | 18e | D; E | Provide details of the availability of the study data | Methods—Data and Code Availability: MIMIC-IV is available through PhysioNet to credentialed users. |
|  | Code sharing | 18f | D; E | Provide details of the availability of the analytical code | Methods—Data and Code Availability: analysis code will be deposited on GitHub; the repository URL remains blank and must be inserted before submission. |
| PATIENT & PUBLIC INVOLVEMENT | Patient & Public Involvement | 19 | D; E | Provide details of any patient and public involvement during the design, conduct, reporting, interpretation, or dissemination of the study or state no involvement. | No patient or public involvement was undertaken for this retrospective de-identified database study; add this statement to the final declarations. |
| RESULTS | Participants | 20a | D; E | Describe the flow of participants through the study, including the number of participants with and without the outcome and, if applicable, a summary of the follow-up time. A diagram may be helpful. | Results—Cohort Characteristics and Figure 1: complete participant flow and outcome counts. |
|  | Participants | 20b | D; E | Report the characteristics overall and, where applicable, for each data source or setting, including the key dates, key predictors (including demographics), treatments received, sample size, number of outcome events, follow-up time, and amount of missing data. A table may be helpful. Report any differences across key demographic groups. | Results—Cohort Characteristics; Table 1 and Tables S1/S3: key predictors, treatments, sample sizes, events, and missingness. |
|  | Participants | 20c | E | For model evaluation, show a comparison with the development data of the distribution of important predictors (demographics, predictors, and outcome). | Results—Cohort Characteristics and Table S3: development-versus-evaluation distributions, including species and outcome prevalence. |
|  | Model development | 21 | D; E | Specify the number of participants and outcome events in each analysis (e.g., for model development, hyperparameter tuning, model evaluation) | Results—Model Development and missing-data sensitivity: n = 552/259 and event counts for primary analyses, with complete-case n = 386/206. |
|  | Model specification | 22 | D | Provide details of the full prediction model (e.g., formula, code, object, application programming interface) to allow predictions in new individuals and to enable third-party evaluation and implementation, including any restrictions to access or re-use (e.g., freely available, proprietary) | Table S16 and code availability: complete Elastic Net coefficients, preprocessing means/SDs, coding, and comparator definitions; fitted preprocessing object is required for exact use. |
|  | Model performance | 23a | D; E | Report model performance estimates with confidence intervals, including for any key subgroups (e.g., sociodemographic). Consider plots to aid presentation. | Results—Model Performance/Calibration/Sensitivity Analyses; Figures 2–3 and Tables S2, S7–S13: estimates with CIs and age/sex subgroup performance. |
|  | Model performance | 23b | D; E | If examined, report results of any heterogeneity in model performance across clusters. See TRIPOD-Cluster for additional details. | Not applicable: no multicenter or other cluster-level heterogeneity analysis. |
|  | Model updating | 24 | E | Report the results from any model updating, including the updated model and subsequent performance | No model updating was performed; the post hoc recalibration trigger and decision not to recalibrate are reported. |
| DISCUSSION | Interpretation | 25 | D; E | Give an overall interpretation of the main results, including issues of fairness in the context of the objectives and previous studies | Discussion—Principal Findings, Comparison with Existing Literature, and Clinical Implications: overall interpretation, calibration, intended use, and limited fairness evidence. |
|  | Limitations | 26 | D; E | Discuss any limitations of the study (such as a non-representative sample, sample size, overfitting, missing data) and their effects on any biases, statistical uncertainty, and generalizability | Discussion—Limitations: single center, selection/overfitting, missing data, species reporting, anchor-year resolution, calibration, unavailable genotype/MALDI-TOF data, and DCA assumptions. |
|  | Usability of the model in the context of current care | 27a | D | Describe how poor quality or unavailable input data (e.g., predictor values) should be assessed and handled when implementing the prediction model | Methods—Missing Data and Preprocessing and Discussion—Transferability: handling unavailable values requires the fitted preprocessing pipeline and local validation. |
|  | Usability of the model in the context of current care | 27b | D | Specify whether users will be required to interact in the handling of the input data or use of the model, and what level of expertise is required of users | Discussion—Clinical Implications: clinician-facing risk stratification only; no stand-alone treatment rule or automated action. |
|  | Usability of the model in the context of current care | 27c | D; E | Discuss any next steps for future research, with a specific view to applicability and generalizability of the model | Discussion—Transferability and Next Steps and Conclusion: multicenter external evaluation, local recalibration/updating, and prospective impact evaluation. |

**Table S18. TRIPOD+AI for Abstracts reporting checklist.**

Reporting locations refer to the manuscript title and Abstract (p. 2). The checklist follows the 13-item TRIPOD+AI for Abstracts guidance.

| **Section** | **Item** | **TRIPOD+AI for Abstracts recommendation** | **Location in this study** |
| --- | --- | --- | --- |
| TITLE | 1 | Identify model development/evaluation, target population, and predicted outcome. | Abstract—Title, p. 2: Title identifies development and temporal evaluation, ICU-onset sterile-site Enterococcus-positive cultures, and vancomycin resistance. |
| BACKGROUND | 2 | Explain the healthcare context and rationale for developing or evaluating the models. | Abstract—Background, p. 2: Introduction: pre-susceptibility decision window and treatment-versus-stewardship trade-off. |
| OBJECTIVES | 3 | Specify whether the study describes model development, evaluation, or both. | Abstract—Objectives, p. 2: Introduction: develop and temporally evaluate a vancomycin-resistance model. |
| METHODS | 4 | Describe the sources of data. | Abstract—Methods, p. 2: Methods: retrospective MIMIC-IV cohort. |
|  | 5 | Describe eligibility criteria and the setting where data were collected. | Abstract—Methods, p. 2: Methods: adult ICU admissions; sterile-site Enterococcus-positive culture obtained >48 hours after ICU admission. |
|  | 6 | Specify the predicted outcome, including the prediction horizon for prognostic models. | Abstract—Methods, p. 2: Introduction/Methods: vancomycin resistance of the index isolate before incorporation of AST; no prognostic horizon. |
|  | 7 | Specify model type, key model-building steps, and internal validation. | Abstract—Methods, p. 2: Methods: Elastic Net selection with bootstrap stability screening; four tree comparators; development-only procedures. |
|  | 8 | Specify model-performance measures. | Abstract—Methods, p. 2: Methods: discrimination, calibration, and exploratory decision-curve performance. |
| RESULTS | 9 | Report the number of participants and outcome events. | Abstract—Results, p. 2: Results: 811 admissions, including 507 VRE; development n=552 and temporal evaluation n=259. |
|  | 10 | Summarize predictors in the final model. | Abstract—Results, p. 2: Methods: 19 predictors spanning species, specimen source, severity, treatment exposures, procedures, and laboratory measurements. |
|  | 11 | Report model-performance estimates with confidence intervals. | Abstract—Results, p. 2: Results: AUROC with 95% CI, calibration slope/CITL, comparator AUROCs, and threshold sensitivity/specificity. |
| DISCUSSION | 12 | Give an overall interpretation of the main results. | Abstract—Discussion, p. 2: Discussion: no statistically detectable tree-model advantage within the common selected set; three models showed under-dispersion; no stand-alone treatment use. |
| REGISTRATION | 13 | Give the registration number and registry/repository name. | Abstract—Registration, p. 2: Registration: not registered. |

## Supplementary Table S19. Sensitivity of model performance to species-level identification

| **Analysis** | **N** | **Events** | **AUROC (95% CI)** | **AUPRC (95% CI)** | **Calibration slope (95% CI)** | **CITL (95% CI)** | **ECE** | **Brier (95% CI)** | **ΔAUROC** | **DeLong P** |
| --- | --- | --- | --- | --- | --- | --- | --- | --- | --- | --- |
| Full 19-predictor model (original held-out predictions) | 259 | 172 | 0.835 (0.778–0.889) | 0.895 (0.848–0.939) | 0.910 (0.710–1.184) | 0.099 (-0.225–0.431) | 0.076 | 0.144 (0.116–0.170) |  |  |
| Genus-level model excluding species (17 predictors) | 259 | 172 | 0.657 (0.588–0.732) | 0.781 (0.715–0.853) | 0.571 (0.320–0.902) | 0.236 (-0.048–0.546) | 0.093 | 0.217 (0.193–0.239) | 0.178 | <0.001 |

*AUROC, area under the receiver operating characteristic curve; AUPRC, area under the precision–recall curve; CITL, calibration-in-the-large; ECE, expected calibration error. Confidence intervals were obtained by paired bootstrap resampling; the AUROC comparison used the paired DeLong test.*

## Supplementary Table S20. Sensitivity analysis forcing time to culture into the model

***Panel A. Predictive performance***

| **Analysis** | **N** | **Events** | **AUROC (95% CI)** | **AUPRC (95% CI)** | **Calibration slope (95% CI)** | **CITL (95% CI)** | **ECE** | **Brier (95% CI)** | **ΔAUROC** | **DeLong P** |
| --- | --- | --- | --- | --- | --- | --- | --- | --- | --- | --- |
| Original 19-predictor model | 259 | 172 | 0.835 (0.778–0.889) | 0.895 (0.848–0.939) | 0.910 (0.710–1.184) | 0.099 (-0.225–0.431) | 0.076 | 0.144 (0.116–0.170) |  |  |
| 20-predictor model including time to culture | 259 | 172 | 0.836 (0.779–0.889) | 0.897 (0.853–0.940) | 0.912 (0.713–1.177) | 0.102 (-0.221–0.434) | 0.075 | 0.144 (0.116–0.170) | -0.001 | 0.364 |

*The 20-predictor sensitivity model forced standardized time to culture into the fixed Elastic Net architecture. CIs were obtained by paired bootstrap resampling.*

***Panel B. Coefficient comparison***

| **Variable** | **β (19-predictor)** | **OR (19-predictor)** | **β (time-adjusted)** | **OR (time-adjusted; 95% CI)** | **β change, %** |
| --- | --- | --- | --- | --- | --- |
| nephrotoxic agents | 0.438 | 1.549 | 0.441 | 1.555 (1.076–2.312) | 0.900 |
| rifamycin | 0.457 | 1.580 | 0.447 | 1.564 (1.003–2.389) | -2.200 |
| corticosteroids | 0.245 | 1.278 | 0.226 | 1.254 (0.881–1.972) | -7.700 |
| endoscopic procedure | 0.329 | 1.390 | 0.300 | 1.350 (0.994–1.893) | -8.900 |
| renal replacement therapy procedure | 0.274 | 1.315 | 0.262 | 1.299 (0.899–2.010) | -4.500 |
| percutaneous drainage | 0.158 | 1.171 | 0.156 | 1.169 (0.878–1.686) | -1.100 |
| Time to culture hours |  |  | 0.075 | 1.078 (0.868–1.511) |  |

*β denotes the fitted log-odds coefficient. OR, odds ratio; CI, confidence interval.*

## Supplementary Table S21. Development-OOF-fitted recalibration

| **Model** | **Method** | **AUROC** | **AUPRC** | **Calibration slope (95% CI)** | **Calibration intercept** | **CITL (95% CI)** | **ECE** | **Brier (95% CI)** |
| --- | --- | --- | --- | --- | --- | --- | --- | --- |
| Elastic Net Logistic | Original | 0.835 | 0.895 | 0.910 (0.710–1.184) | 0.139 | 0.099 (-0.225–0.431) | 0.076 | 0.144 (0.116–0.170) |
| Elastic Net Logistic | Platt | 0.835 | 0.895 | 0.827 (0.645–1.076) | 0.173 | 0.095 (-0.259–0.447) | 0.069 | 0.144 (0.115–0.171) |
| Elastic Net Logistic | Isotonic | 0.834 | 0.884 | 0.307 (0.135–0.836) | 0.370 | 0.101 (-0.285–0.522) | 0.044 | 0.144 (0.115–0.173) |
| Random Forest | Original | 0.848 | 0.889 | 1.414 (1.079–1.873) | -0.099 | 0.105 (-0.149–0.380) | 0.079 | 0.138 (0.115–0.162) |
| Random Forest | Platt | 0.848 | 0.889 | 0.942 (0.719–1.249) | 0.060 | 0.028 (-0.304–0.353) | 0.067 | 0.131 (0.103–0.160) |
| Random Forest | Isotonic | 0.848 | 0.885 | 0.483 (0.144–1.119) | 0.408 | 0.077 (-0.259–0.420) | 0.046 | 0.128 (0.099–0.157) |
| XGBoost | Original | 0.842 | 0.877 | 2.415 (1.876–3.159) | -0.514 | 0.145 (-0.075–0.406) | 0.153 | 0.158 (0.140–0.175) |
| XGBoost | Platt | 0.842 | 0.877 | 1.024 (0.796–1.339) | 0.045 | 0.057 (-0.250–0.372) | 0.066 | 0.132 (0.105–0.160) |
| XGBoost | Isotonic | 0.843 | 0.875 | 0.414 (0.141–1.171) | 0.515 | 0.129 (-0.185–0.463) | 0.048 | 0.132 (0.104–0.160) |
| LightGBM | Original | 0.853 | 0.904 | 1.075 (0.834–1.412) | 0.002 | 0.043 (-0.257–0.352) | 0.054 | 0.133 (0.108–0.160) |
| LightGBM | Platt | 0.853 | 0.904 | 1.067 (0.828–1.401) | 0.027 | 0.061 (-0.240–0.373) | 0.056 | 0.133 (0.107–0.160) |
| LightGBM | Isotonic | 0.852 | 0.886 | 0.954 (0.747–1.231) | 0.173 | 0.146 (-0.168–0.489) | 0.031 | 0.134 (0.106–0.161) |
| CatBoost | Original | 0.855 | 0.894 | 1.462 (1.140–1.880) | -0.085 | 0.139 (-0.118–0.410) | 0.082 | 0.133 (0.111–0.156) |
| CatBoost | Platt | 0.855 | 0.894 | 1.008 (0.786–1.296) | 0.027 | 0.032 (-0.294–0.363) | 0.057 | 0.126 (0.100–0.156) |
| CatBoost | Isotonic | 0.852 | 0.880 | 0.998 (0.783–1.283) | 0.049 | 0.048 (-0.278–0.375) | 0.022 | 0.125 (0.097–0.154) |

*Platt and isotonic recalibrators were fitted exclusively to development-set out-of-fold predictions and applied unchanged to pseudo-temporal validation predictions. CITL, calibration-in-the-large; ECE, expected calibration error.*

## Supplementary Table S22. Descriptive recorded race/ethnicity subgroup performance

| **Dataset** | **Recorded group** | **N** | **Events** | **Event rate** | **AUROC** | **AUPRC** | **Interpretive caveat** |
| --- | --- | --- | --- | --- | --- | --- | --- |
| Development | Recorded White | 378 | 234 | 0.619 | 0.848 | 0.887 | Descriptive; not a fairness-equivalence test |
| Development | Recorded non-White | 174 | 101 | 0.580 | 0.807 | 0.831 | Descriptive; not a fairness-equivalence test |
| Development | Recorded Black | 66 | 36 | 0.545 | 0.825 | 0.834 | Descriptive; not a fairness-equivalence test |
| Development | Recorded Hispanic | 27 | 16 | 0.593 | 0.847 | 0.916 | Descriptive; not a fairness-equivalence test |
| Development | Recorded Asian | 21 | 14 | 0.667 | 0.827 | 0.897 | Descriptive; not a fairness-equivalence test |
| Development | Recorded other/unknown | 60 | 35 | 0.583 | 0.755 | 0.770 | Descriptive; not a fairness-equivalence test |
| Pseudo-temporal validation | Recorded White | 151 | 97 | 0.642 | 0.819 | 0.874 | Descriptive; not a fairness-equivalence test |
| Pseudo-temporal validation | Recorded non-White | 108 | 75 | 0.694 | 0.864 | 0.924 | Descriptive; not a fairness-equivalence test |
| Pseudo-temporal validation | Recorded Black | 17 | 8 | 0.471 | 0.819 | 0.732 | Descriptive only; too small for fairness conclusions |
| Pseudo-temporal validation | Recorded Hispanic | 13 | 11 | 0.846 | 1.000 | 1.000 | Descriptive only; too small for fairness conclusions |
| Pseudo-temporal validation | Recorded Asian | 15 | 11 | 0.733 | 1.000 | 1.000 | Descriptive only; too small for fairness conclusions |
| Pseudo-temporal validation | Recorded other/unknown | 63 | 45 | 0.714 | 0.822 | 0.919 | Descriptive; not a fairness-equivalence test |

*Race and ethnicity reflect recorded database categories. Results are descriptive and are not fairness-equivalence tests; very small subgroups preclude reliable conclusions.*

## Supplementary Table S23. Selection-inclusive bootstrap optimism correction

| **Analysis** | **Requested outer resamples** | **Successful outer resamples** | **Inner stability resamples** | **Mean optimism (95% empirical interval)** | **Selected predictors, median (IQR)** | **Note** |
| --- | --- | --- | --- | --- | --- | --- |
| Selection-inclusive bootstrap | 100 | 100 | 100 | 0.040 (0.010–0.067) | 17 (15–19) | Fixed selector and final-model hyperparameters; preprocessing matrix fixed; selection and fitting repeated. |

*The outer bootstrap repeated support screening, fixed-hyperparameter stability selection, feature capping, and final fitting. It did not repeat every preprocessing or hyperparameter-tuning decision.*

## Supplementary Table S24. Aspartate aminotransferase drop and imputation-direction sensitivity

***Panel A. Performance after removing aspartate aminotransferase***

| **Analysis** | **N** | **Events** | **AUROC (95% CI)** | **AUPRC (95% CI)** | **Calibration slope (95% CI)** | **CITL (95% CI)** | **ECE** | **Brier (95% CI)** | **ΔAUROC** | **DeLong P** |
| --- | --- | --- | --- | --- | --- | --- | --- | --- | --- | --- |
| Original 19-predictor model | 259 | 172 | 0.835 (0.778–0.889) | 0.895 (0.848–0.939) | 0.910 (0.710–1.184) | 0.099 (-0.225–0.431) | 0.076 | 0.144 (0.116–0.170) |  |  |
| 18-predictor model excluding aspartate aminotransferase | 259 | 172 | 0.845 (0.788–0.896) | 0.902 (0.857–0.944) | 0.990 (0.764–1.299) | 0.062 (-0.239–0.381) | 0.074 | 0.138 (0.112–0.166) | -0.010 | 0.099 |

*CIs were obtained by paired bootstrap resampling; the AUROC comparison used the paired DeLong test.*

***Panel B. Direction of the aspartate aminotransferase coefficient across imputation strategies***

| **Imputation strategy** | **β** | **OR** | **β range** | **OR range** |
| --- | --- | --- | --- | --- |
| Iterative | -0.284 | 0.753 |  |  |
| Median | -0.268 | 0.765 |  |  |
| Posterior-draw iterative imputation (10 datasets) | -0.179 | 0.836 | -0.272 to -0.087 | 0.762–0.916 |

*β denotes the fitted log-odds coefficient; OR, odds ratio.*

## Supplementary Table S25. Decision-curve net benefit with paired bootstrap confidence intervals

| **Model** | **Threshold** | **Net benefit (95% CI)** | **Difference vs treat-all (95% CI)** |
| --- | --- | --- | --- |
| Elastic Net Logistic | 0.100 | 0.620 (0.557–0.685) | -0.007 (-0.023–0.005) |
| Elastic Net Logistic | 0.200 | 0.584 (0.513–0.655) | 0.004 (-0.018–0.023) |
| Elastic Net Logistic | 0.300 | 0.564 (0.491–0.637) | 0.044 (0.014–0.071) |
| Elastic Net Logistic | 0.500 | 0.479 (0.401–0.560) | 0.151 (0.085–0.212) |
| Random Forest | 0.100 | 0.627 (0.562–0.683) | 0.000 (0.000–0.000) |
| Random Forest | 0.200 | 0.579 (0.503–0.648) | -0.001 (-0.016–0.013) |
| Random Forest | 0.300 | 0.555 (0.482–0.628) | 0.035 (0.012–0.059) |
| Random Forest | 0.500 | 0.490 (0.401–0.575) | 0.162 (0.100–0.224) |
| XGBoost | 0.100 | 0.627 (0.562–0.695) | 0.000 (0.000–0.000) |
| XGBoost | 0.200 | 0.580 (0.503–0.648) | 0.000 (0.000–0.000) |
| XGBoost | 0.300 | 0.516 (0.430–0.601) | -0.004 (-0.012–0.000) |
| XGBoost | 0.500 | 0.494 (0.413–0.579) | 0.166 (0.112–0.224) |
| LightGBM | 0.100 | 0.619 (0.561–0.682) | -0.008 (-0.022–0.004) |
| LightGBM | 0.200 | 0.587 (0.524–0.656) | 0.007 (-0.013–0.026) |
| LightGBM | 0.300 | 0.568 (0.495–0.636) | 0.048 (0.019–0.075) |
| LightGBM | 0.500 | 0.494 (0.413–0.579) | 0.166 (0.108–0.228) |
| CatBoost | 0.100 | 0.627 (0.567–0.687) | 0.000 (0.000–0.000) |
| CatBoost | 0.200 | 0.587 (0.519–0.651) | 0.007 (-0.010–0.021) |
| CatBoost | 0.300 | 0.562 (0.486–0.632) | 0.042 (0.017–0.067) |
| CatBoost | 0.500 | 0.506 (0.425–0.579) | 0.178 (0.116–0.243) |
| Treat all | 0.100 | 0.627 (0.562–0.687) |  |
| Treat all | 0.200 | 0.580 (0.508–0.648) |  |
| Treat all | 0.300 | 0.520 (0.432–0.597) |  |
| Treat all | 0.500 | 0.328 (0.220–0.444) |  |
| Treat none | 0.100 | 0.000 (0.000–0.000) |  |
| Treat none | 0.200 | 0.000 (0.000–0.000) |  |
| Treat none | 0.300 | 0.000 (0.000–0.000) |  |
| Treat none | 0.500 | 0.000 (0.000–0.000) |  |

*Net benefit and model-minus-treat-all differences were estimated with 1,000 paired validation-set bootstrap resamples. Treat-all and treat-none are mathematical reference strategies, not observed clinical policies.*

## Supplementary Table S26. Performance by anchor-year group

| **Model** | **Anchor-year group** | **N** | **Events** | **Event rate** | **AUROC (95% CI)** | **AUPRC (95% CI)** | **Calibration slope (95% CI)** | **CITL (95% CI)** | **Brier (95% CI)** |
| --- | --- | --- | --- | --- | --- | --- | --- | --- | --- |
| Elastic Net Logistic | 2017 - 2019 | 150 | 95 | 0.633 | 0.808 (0.732–0.884) | 0.859 (0.786–0.929) | 0.855 (0.579–1.290) | 0.003 (-0.384–0.415) | 0.160 (0.121–0.200) |
| LightGBM | 2017 - 2019 | 150 | 95 | 0.633 | 0.829 (0.755–0.900) | 0.877 (0.811–0.938) | 0.968 (0.674–1.439) | -0.099 (-0.499–0.302) | 0.153 (0.116–0.191) |
| Elastic Net Logistic | 2020 - 2022 | 109 | 77 | 0.706 | 0.863 (0.775–0.933) | 0.932 (0.879–0.972) | 0.977 (0.676–1.508) | 0.260 (-0.254–0.850) | 0.121 (0.084–0.163) |
| LightGBM | 2020 - 2022 | 109 | 77 | 0.706 | 0.888 (0.806–0.950) | 0.946 (0.899–0.980) | 1.226 (0.832–1.998) | 0.269 (-0.195–0.758) | 0.107 (0.073–0.147) |

*Anchor-year groups are de-identified database groups and should not be interpreted as literal calendar-time strata. Results are descriptive.*

**Supplementary Figures**

**Figure S1. Temporal trend in VRE prevalence by anchor year group.**

Vancomycin-resistant *Enterococcus* (VRE) prevalence (point estimate) by three-year anchor-year group, with 95% Wilson confidence intervals. The vertical dashed red line indicates the split between anchor-year groups labelled 2008–2016 (development) and 2017–2022 (pseudo-temporal validation). VRE prevalence increased across the groups, with the highest estimates in groups labelled 2020–2022.

**Figure S2. Bootstrap selection frequency for candidate predictors with non-zero Elastic Net coefficients.**

Bootstrap selection frequency (proportion of 100 resamples of the development set in which the predictor received a non-zero Elastic Net Logistic coefficient) for all 45 candidate predictors with a non-zero selection frequency. Predictors above the dashed line (frequency ≥ 0.50; red bars) met the prespecified stability threshold; those below the threshold are shown in grey. Among the 28 stable predictors, nine failed the development-set support criteria and were excluded; 19 predictors were retained for final modeling.

**Figure S3. Precision-recall curves on the pseudo-temporal validation set.**

Precision-recall (PR) curves for the five candidate models on the pseudo-temporal validation set (n = 259). Average precision (AP, equivalent to AUPRC) is shown in the legend for each model. The dashed horizontal line indicates the no-discrimination baseline equal to the validation-set VRE prevalence (0.664). All models substantially exceed the prevalence baseline across the full recall range.

**Figure S4. Forest plot of AUROC with 95% bootstrap confidence intervals on the pseudo-temporal validation set.**

Point estimates and 95% bootstrap confidence intervals (1,000 paired resamples) for AUROC across the five candidate models on the pseudo-temporal validation set (n = 259). The vertical dashed line marks the no-discrimination reference (AUROC = 0.50). Confidence intervals overlap substantially across models, consistent with the non-significant DeLong tests (see Table S7).

**Figure S5. Spearman rank correlation matrix of the 19 final predictors.**

Spearman rank correlation matrix for the 19 final predictors in the development set (n = 552). Colour intensity indicates the magnitude and direction of correlation (red, positive; blue, negative). Pairwise correlations were predominantly weak to moderate; the strongest negative correlation occurred between *Enterococcus faecium* and *Enterococcus faecalis*, as expected from their one-hot encoding from the same categorical variable.

## Supplementary Figure S6. Recalibration of under-dispersed tree models

Original, Platt-scaled, and isotonic calibration curves for Random Forest, XGBoost, and CatBoost in the pseudo-temporal validation set. Both recalibrators were fitted exclusively to development-set out-of-fold predictions and applied unchanged to validation predictions.
